# Ferritin and transferrin predict common carotid intima-media thickness in females: a machine-learning informed individual participant data meta-analysis

**DOI:** 10.1186/s12872-026-05796-8

**Published:** 2026-04-14

**Authors:** Anand Ruban Agarvas, Richard Sparla, Janice L Atkins, Claudia Altamura, Todd Anderson, Ebru Asicioglu, Judit Bassols, Abel López-Bermejo, Hana Marie Dvořáková, José Manuel Fernández-Real, Christoph Hochmayr, Michael Knoflach, Jovana Kusic Milicevic, Silvia Lai, José María Moreno-Navarrete, Dariusz Pawlak, Krystyna Pawlak, Petr Syrovatka, Dorota Formanowicz, Pavel Kraml, Jose M Valdivielso, Luca Valenti, Martina U. Muckenthaler

**Affiliations:** 1 Present Address: Spinal Cord Injury Center, Heidelberg University Hospital, Heidelberg University, Heidelberg, Germany; 2 Center For Translational Biomedical Iron Research, Department of Pediatric Hematology, Oncology Immunology and Pulmonology, Heidelberg University Hospital, Heidelberg, Germany; 3 Department of Clinical & Biomedical Sciences, Faculty of Health & Life Sciences, University of Exeter, Exeter, UK; 4 Unit of Headache and Neurosonology, Department of Medicine and Surgery, Università Campus Bio-Medico di Roma, Rome, Italy; 5 Department of Cardiac Sciences and Libin Cardiovascular Institute of Alberta (TJA), Cumming School of Medicine, University of Calgary, Calgary, AB Canada; 6 Pendik Training and Research Hospital, Marmara University, Istanbul, Turkey; 7 Institut d’Investigació Biomèdica de Girona (IdIBGi), Girona, Spain; 8 Department of Neonatology, University Hospital Prague-Motol, Prague, Czech Republic; 9 Hospital Trueta & Department of Medical Sciences, School of Medicine, Institut d’Investigació Biomèdica de Girona (IdIBGi), Universitat de Girona, Girona, Spain; 10 CIBERobn Fisiopatologia de la Obesidad y Nutrición, Madrid, Spain; 11 Department of Pediatrics II (Neonatology), Medical University of Innsbruck, Innsbruck, Austria; 12 Department of Neurology, Medical University of Innsbruck, Innsbruck, Austria; 13 VASCage, Centre on Clincial Stroke Research, Innsbruck, Austria; 14 Department of Nephrology, Clinical Hospital Centre Zemun, Faculty of Medicine, University of Belgrade, Belgrade, Serbia; 15 Department of Translational and Precision Medicine, Nephrology Unit, Sapienza University of Rome, Rome, Italy; 16 Department of Pharmacodynamics, Medical University of Bialystok, Bialystok, Poland; 17 Department of Monitored Pharmacotherapy, Medical University of Bialystok, Bialystok, Poland; 18 Cardiocentre, Institute for Clinical and Experimental Medicine, Prague, Czech Republic; 19 Department of Medical Chemistry and Laboratory Medicine, Poznan University of Medical Sciences, Poznan, Poland; 20 Department of Medicine, Third Faculty of Medicine Charles University and University Hospital Královské Vinohrady, Prague, Czech Republic; 21 Vascular and Renal Translational Research Group, IRBLleida, Lleida, Spain; 22 Department of Pathophysiology and Transplantation, Università degli Studi di Milano, Milano, Italy; 23 Precision Medicine, SS Centro Risorse Biologiche, Fondazione IRCCS Ca’ Granda Policlinico, Pad Marangoni via F Sforza 35, Milano, 20122 Italy; 24 Molecular Medicine Partnership Unit, Heidelberg, Germany; 25 Translational Lung Research Center Heidelberg (TLRC), German Center for Lung Research (DZL), Heidelberg, Germany; 26 German Centre for Cardiovascular Research (DZHK), Partner Site Heidelberg/Mannheim, Heidelberg, Germany

**Keywords:** Carotid Artery Diseases, Atherosclerosis, Iron, Hyperferritinemia, Female

## Abstract

**Background:**

Iron overload promotes atherosclerosis in mice and causes vascular dysfunction in humans with Hemochromatosis. However, data are controversial on whether systemic iron availability within physiological limits affects the pathogenesis of atherosclerosis. We, therefore, performed an individual participant data (IPD) meta-analysis and studied the association between serum iron biomarkers with common carotid intima-media thickness (CC-IMT); in addition, since sex influences iron metabolism and vascular diseases, we studied if there are sex-specific differences.

**Methods:**

We pooled the IPD and analysed the data on adults (age≥18y) by orthogonal approaches: machine learning (ML) and a single-stage meta-analysis. For ML, we tuned a gradient-boosted tree regression model (XGBoost) and subsequently, we interpreted the features using variable importance. For the single-stage metaanalysis, we examined the association between iron biomarkers and CC-IMT using spline-based linear mixed models, accounting for sex interactions and study-specific effects. To confirm robustness, we repeated analyses on imputed data using multivariable regression adjusted for key covariates identified through machine learning. Further, subgroup analyses were performed in children and adolescents (age<18y). In addition, to evaluate causality, we used UK Biobank data to examine associations between the hemochromatosis (HFE) genotypes (C282Y/H63D) and mean CC-IMT in ~ 42,500 participants with carotid ultrasound data, using sex-stratified linear regression (adjusted for age, assessment centre, and genetic principal components).

**Results:**

We included IPD from 21 studies (*N* = 10,807). The application of the ML model showed moderate predictive performance and identified iron biomarkers (transferrin, ferritin, transferrin saturation, and iron) as key features for IMT prediction. Multivariable analyses showed non-linear sex-specific relationships for ferritin and transferrin with CC-IMT, both only among females at specific ranges. Ferritin showed a significant positive association [Ferritin > 233 ng/mL: β = 0.04, 95% CI (0.002, 0.08), *p* = 0.037], while transferrin showed negative associations at specific ranges [ Transferrrin 231–263 mg/dL: β=-0.21, 95% CI (-0.43, 0.003), *p* = 0.054; Transferrrin > 263 mg/dL: β=-0.73, 95% CI (-1.48, 0.01), *p* = 0.05]; No significant associations were found between CC-IMT in those with HFE genotypes in either sex in the UK Biobank.

**Conclusion:**

Our observational data show that iron biomarkers - ferritin and transferrin are non-linearly associated with CC-IMT specifically in females, while a significant causal association between the HFE genotype and CC-IMT could not be demonstrated in the UK Biobank data. We conclude that our observational findings may reflect residual confounding, reverse causation, or other non-causal mechanisms rather than a direct causal relationship.

**Other:**

No financial support was received for this meta-analysis. The protocol for this study is registered in the PROSPERO database ( CRD42020155429; https://www.crd.york.ac.uk/).

**Supplementary Information:**

The online version contains supplementary material available at 10.1186/s12872-026-05796-8.

## Introduction

Iron is a vital nutrient involved in numerous cellular functions; however, iron accumulation can be pathological. We have previously shown that excess iron accelerates the pathogenesis of atherosclerosis [1]. Excess iron initiates redox reactions (via Fenton chemistry) that lead to oxidative damage of membrane lipids, proteins, and DNA. In addition, iron can also lead to endothelial injury, immune cell polarization, ferroptosis, and plaque destabilization, thereby, contributing to vascular dysfunction and atherosclerosis [1–3]. In the blood, iron biomarkers reflective of the systemic iron status [e.g., serum iron, ferritin, transferrin, transferrin saturation (TSAT), total iron binding capacity (TIBC) and hepcidin] can be quantified. Whether iron biomarkers within physiological ranges are associated with vascular disease is clinically relevant, but existing evidence is conflicting.

Sonographic assessment of common carotid intima–media thickness (CC-IMT) reflects arteriopathy and independently predicts cardiovascular events [4, 5], making it a suitable surrogate marker of vascular disease. To clarify the relationship between iron biomarkers and vascular pathology, and in light of inconsistent findings from previous smaller studies, we conducted an individual participant data (IPD) meta-analysis. Further, we wanted to investigate the influence of sex on the association between iron biomarkers and CC-IMT since sex-specific differences in cardiovascular disease [6] and iron metabolism [7, 8] are commonly observed. Specifically, the female sexual hormones estrogen and progesterone impair endothelial function and affect iron homeostasis by controlling hepcidin, the master regulator of iron homeostasis [9, 10]. Our recent analysis in two cohorts also found a positive association between ferritin and peripheral arterial disease preferentially in females, suggesting sex-specific effects [11]. In addition, our current study additionally aimed to explore early vascular changes across a broader age spectrum. For example, in autopsies, atherosclerotic fibrous plaques have been detected in the aorta and coronary artery participants between 2 and 15 years [12]. Therefore, we included children and adolescents in subgroup analyses and analyzed them separately from adults since CC-IMT is strongly influenced by age.

We incorporated two complementary analytical strategies in the meta-analysis—traditional regression modeling and machine learning (ML)—each providing distinct advantages and limitations for identifying patterns and sources of heterogeneity in the data. The idea to combine machine learning with traditional approaches is based on concepts proposed previously (for example [13]. Integration of ML approaches with regression-based analyses is an active area of research [13, 14], which is still being methodologically optimized. Machine learning is well suited for analyzing complex, high-dimensional datasets, enabling the identification of potential nonlinear relationships [15, 16]. On the other hand, regression analysis enables testing of relationships between variables under specific assumptions. Therefore, we aimed to combine the strengths of predictive modelling with the interpretability and inferential capacity of traditional statistical methods. We believe that systematically combining methods with different strength offers an exciting avenue for improving both prediction and mechanistic understanding. Such integrative strategies may help bridge the gap between complex data and clinically interpretable insights, and we anticipate that these hybrid approaches will become increasingly important in future medical research. Our choice to consider nonlinear relationships was informed by our recent paper on iron and peripheral arterial disease [11]. Iin addition, there are also several other studies that have demonstrated U‑shaped relationships between various risk factors and cardiovascular risk [17]. Finally, to complement the observational analyses of the iron biomarkers, we performed a Mendelian randomization (MR) to assess the potential causal relationship between hemochromatosis (HFE) genotypes (C282Y/H63D) and CC-IMT in the UK Biobank data.

## Methods

**Fig. 1:**
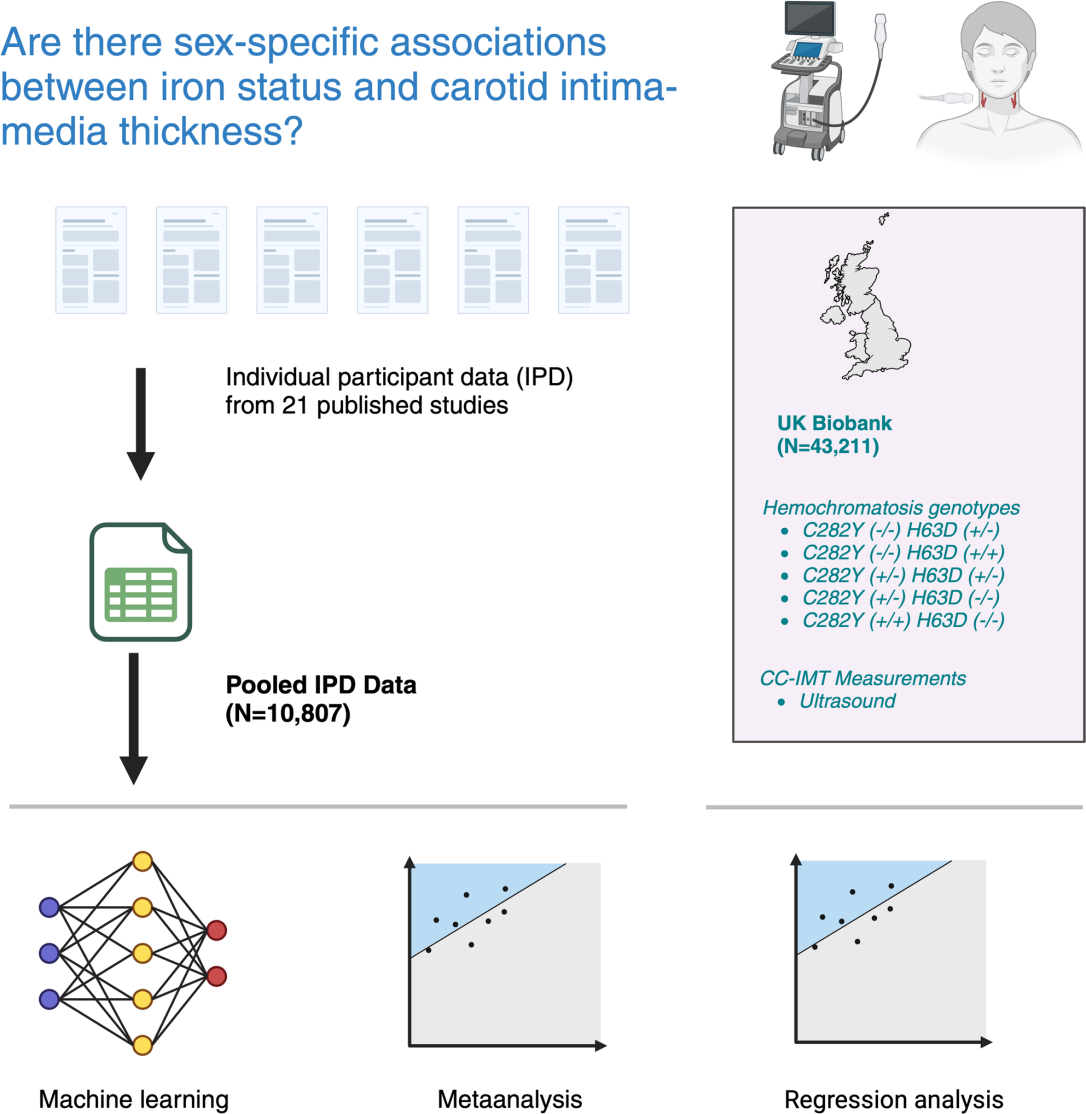
The outline of the study and analysis

An outline of the study is shown in Fig. 1. The protocol for the study is published in the PROSPERO database (CRD42020155429; https://www.crd.york.ac.uk/; Supplementary File 1). We report the study according to Preferred Reporting Items for Systematic Review and Meta-Analyses of individual participant data (PRISMA-IPD; See Supplementary File 2 for checklist [18]). We used R version 4.3.1 [19] for the data analysis and visualization (For the specific packages used, see Supplementary Table 1).

### Literature search

We searched NLM Medline using the following string: (iron OR ferritin OR transferrin OR hepcidin) AND (atherosclerosis OR intima-media thickness). We applied filters for human studies published in English between 1st Oct 1999 and 20th Oct 2019 (last updated on 24th Aug 2023). We used a three-step process for the study selection. In step 1, we screened the titles of the published studies and excluded records when the titles were specified as reviews or were performed on preclinical models. In step 2, we screened the abstract for human studies with one of the following keywords: iron, ferritin, transferrin, hepcidin, atherosclerosis or intima-media thickness. In step 3, we screened the full text and if studies contained data on iron biomarkers [iron, ferritin, transferrin, hepcidin, or transferrin saturation (TSAT)] and CC-IMT, they were included in the IPD. Screening of the retrieved records was done independently by two investigators (ARA, RS).

### Data collection

We contacted the investigators (first or corresponding authors) of eligible studies with the study protocol and requested formal consent for participation (for a scheme of data request, see Supplementary Fig. 1). Through subsequent contact, we asked the investigators for anonymized data on the following variables: age, gender, CC-IMT, serum iron indices (iron, ferritin, transferrin, TSAT, hepcidin), ethnic profile, and presence of comorbidities [e.g., diabetes, hypertension, chronic kidney disease (CKD), hemochromatosis, thalassemia]. For prospective studies, only the baseline data were obtained. We sent three follow-up reminders to investigators who had not responded.

From the studies for which IPD were received, we extracted the data and screened for inclusion in the meta-analysis. We piloted the data extraction by harmonizing the coding of categorical variables (e.g., gender, comorbidities) and their units of measurements, in the case of quantitative variables (Table 1). At this stage, we also compared (variables available in the received datasheets and their original publications) and identified variables common to various studies. When required, we contacted investigators again to request additional variables of interest.

**Table 1:** Characteristics of studies included in the IPD-MA

Study	Participant source	Controls	*N* (%)	Study population
Pawlak et al. [39]	Hospital-based	Yes	26 (0.24%)	Participants with chronic kidney disease on hemodialysis
Hahalis et al. [40]	Hospital-based	Yes	70 (0.65%)	Healthy volunteers and participants with β-thalassemia major
Anderson et al. [41]	Population-based	No	1,578 (14.6%)	Healthy males
Altamura et al. [42]	Hospital-based	Yes	41 (0.38%)	Individuals who had acute stroke and elderly controls without a history of neurological or vascular disease
Risko et al. [43]	Hospital-based	Yes	161 (1.49%)	Healthy individuals and individuals with cardiovascular disease.
Risko et al. [44]	Hospital-based	Yes	161 (1.49%)	Healthy men (long-term blood donors and non-donors).
Syrovatka et al. [45]	Hospital-based	No	161 (1.49%)	Healthy males
Valenti et al. [46]	Hospital-based	No	506 (4.68%)	Participants with nonalcoholic fatty liver disease. In a subset of participants, the presence of C282Y and H63D HFE mutations were analysed.
Prats-Puig et al. [47]	Hospital-based	No	854 (7.9%)	Healthy children
Merchant et al. [48]	Hospital-based	Yes	78 (0.72%)	Participants with β-thalassemia major and controls.
Dvorakova et al. [49]	Hospital-based	Yes	59 (0.55%)	Children with chronic kidney disease and healthy controls
Asicioglu et al.[50]	Hospital-based	Yes	36 (0.33%)	Healthy volunteers and individuals who had a kidney transplant
Galesloot et al. [51]	Population-based	No	1,517 (14.04%)	Participants of the Nijmegen Biomedical study
Arroyo et al. [52]	Hospital-based	Yes	3,004 (27.8%)	Participants with chronic kidney disease and controls with a glomerular filtration rate > 60 ml/min/m2
Formanowicz et al. [53]	Hospital-based	Yes	158 (1.46%)	Healthy controls and individuals with chronic kidney disease
Lai et al. [54]	Hospital-based	Yes	68 (0.63%)	Healthy controls and individuals with chronic kidney disease
Abaza et al. [55]	Hospital-based	Yes	42 (0.39%)	Participants with β-thalassemia major and healthy controls
Kusic Milicevic et al. [56]	Hospital-based	No	60 (0.56%)	Participants with chronic kidney disease on hemodialysis
Bernar et al. [57]	Population-based	No	2,102 (19.45%)	Healthy adolescents
Fernandez-Real et al. [60]	Population-based	No	79 (0.73%)	Healthy volunteers

Table of the characteristics of studies that provided the individual participant data and included in the meta-analysis. The *N* indicates the number of participants per study and the percentage shown are the proportion of the total number of pooled data

**Fig. 2:**
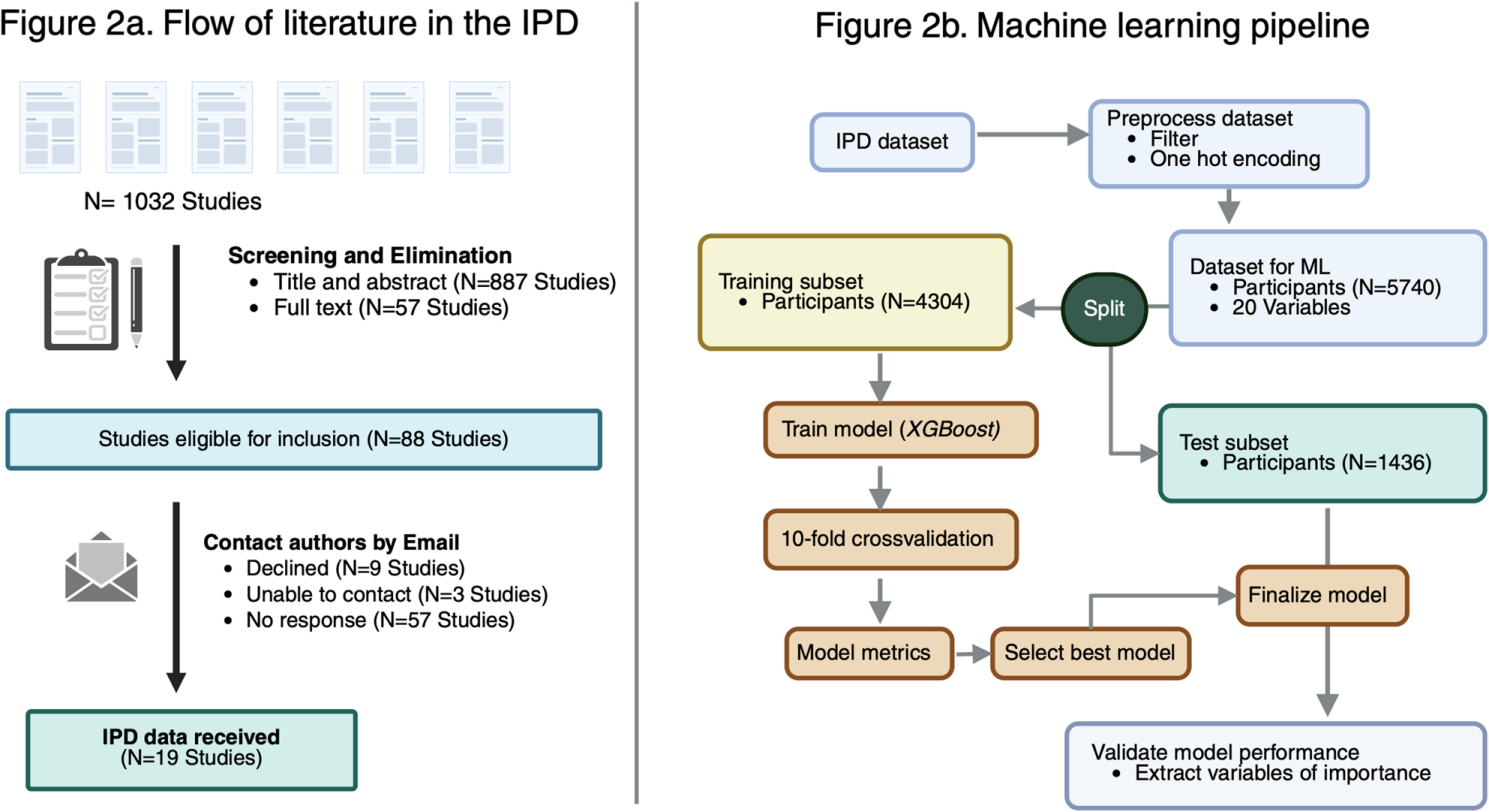
**a** The flow of literature in the IPD. **b** The machine learning pipeline

Subsequently, we reextracted the data and checked the files for data integrity in three steps. In step 1, we compared the number of data participants, sex ratio, and the summary data of variables between the data file and its corresponding original paper. In the step 2, we verified the frequency distribution of continuous variables of interest in the individual data files. If required, we contacted the authors in a third step for clarifications. We excluded studies that did not clear the data integrity check (Fig. 2a).

### Participant-level data

From the received data, all participants with an available CC-IMT measurement were selected for pooling from the selected studies. Next, we selected the commonly measured serum iron biomarkers (iron, ferritin, transferrin, and TSAT) and the demographic and laboratory variables: age, sex, body mass index (BMI), smoking status, presence of comorbidities (diabetes, hypertension, CKD, thalassemia, hemochromatosis), creatinine, hemoglobin, high-density lipoprotein cholesterol (HDLc), low-density lipoprotein cholesterol (LDLc), triacylglycerols, fasting glucose, c-reactive protein (CRP), systolic blood pressure (SBP), and diastolic blood pressure (DBP).

When discrete CC-IMT measurements were available on the right and left carotid arteries, the mean CC-IMT was calculated and used for downstream analyses (without further transformations). The data on age, sex, BMI, and the presence of diabetes, hypertension, CKD, thalassemia, and hemochromatosis were used, as indicated, in the original data files. Since the reporting units of the laboratory variables were not uniform, this required harmonization by conversion factors (e.g., mg/dL to mmol/L). Subsequently, we pooled the IPD and added our own data [11] from 323 individuals from the Heidelberg Study on Diabetes and Complications (HEIST-DiC study; https://clinicaltrials.gov, NCT03022721).

At the participant level, we applied the following exclusion criteria: (1) CC-IMT value suggestive of an atherosclerotic plaque [20] (> 1.5 mm) (2) diagnosed thalassemia or hemochromatosis (3) TSAT and ferritin values suggestive of possibly hemochromatosis [21] and, (4) CRP > 10 mg/dL suggesting overt inflammation [22]. For further curating the ML dataset, stricter inclusion criteria were applied to ensure data completeness and accuracy. Only adult participants (age ≥ 18 years) with valid CC-IMT measurements and at least one iron biomarker (iron, ferritin, transferrin, or TSAT) were included. In the conventional regression models, our aim was to maximize statistical power and generalizability by retaining as many participants as possible. In contrast, the ML analyses required stricter inclusion criteria to ensure high data completeness and accuracy, as model performance is highly sensitive to incomplete or inconsistent inputs.

### Implementation of the ML framework

We converted categorical variables into numerical variables. As the outcome variable (CC-IMT) was continuous and approximately normally distributed, no additional resampling was necessary. The dataset was randomly split into training (75%) and test (25%) sets, stratified by study to preserve proportional representation of individual studies in both partitions. In addition, to avoid information leakage between these subsets, we processed them independently. A preprocessing recipe was created using the recipes package. Predictors were first subjected to bagged tree imputation to address missing values. All numeric predictors were then centered and scaled to zero mean and unit variance. The variable ‘Study’ was treated as an identifier and excluded from model fitting. A gradient-boosted tree model [XGBoost [23] ] was implemented and the following hyperparameters were tuned for the number of trees (15 values sampled via space-filling design), tree depth and learning rate. Hyperparameter tuning was performed via grid search combined with 10-fold cross-validation on the training data. The optimal configuration was selected based on the lowest Root Mean Squared Error (RMSE). After selecting the best hyperparameters based on RMSE, the final model was retrained on the full training set and evaluated on the held-out test set. We then quantified the predictive performance using RMSE and R² metrics.

Model interpretability was assessed using variable importance (VIP) and [SHapley Additive exPlanations [24] ] analyses. The top predictors contributing to model predictions were visualized using the vip and shapviz packages. SHAP values were computed from the fitted XGBoost model and summarized as both importance and dependence plots. The ML pipeline is shown in Fig. 2b, a Glossary of terms pertaining to ML is shown in Box 1 and the pseudocdeis provided in Supplementary file 3.

### Regression analyses

For the regression analyses, since iron and atherosclerosis parameters show age-specific variations, we used data from adults (age≥18y) for the main analysis. Here, we first tested the association between CC-IMT and each of the iron biomarkers (iron, ferritin, transferrin, and TSAT) by linear mixed model regression in the complete (unimputed) data. We hypothesized that the relationship could be nonlinear, and therefore, flexibly modelled the relationship using spline regression (degree of freedom = 4). This choice of four degrees of freedom was prespecified based on established methodological recommendations, which suggest that 3–5 degrees of freedom provide sufficient flexibility to capture biologically plausible non-linear relationships while maintaining model stability [25, 26]. We avoided manually specifying knots since 4 degrees of freedom automatically constrains their placement to a priori quantiles of the biomarker distribution—an approach that ensures robust estimation across the full range without data-driven selection bias. In addition, since iron metabolism shows strong sex-specific differences, we specified it using an interaction term (e.g. ferritin*sex) in the models. This approach is widely accepted in epidemiological and clinical research when exploring whether the association between a predictor (in this case, ferritin, as a proxy for iron metabolism) and an outcome (CC-IMT) differs by a stratifying variable (sex). The use of interaction terms allows for formal statistical testing of effect modification by sex, rather than relying on stratified analyses alone [27]. Further, to account for differences between the different datasets, we included “study” as a random effect in the model.

To further test the robustness of the observations from the previous step, we conducted a complex multivariable regression analysis between CC-IMT and ferritin or transferrin. Here, we imputed the missing values based on the approach by Gibbs sampler [28, 29] and generated multiple imputations (*n* = 5) from a joint multivariable linear-mixed model. For continuous variables, the method considers the relationships between all variables at once, while for categorical variables, the model assumes that they are linked to underlying continuous variables that follow a normal distribution [30]. We performed the regression on the independent imputation draws, using Rubin’s rule [31] and obtained the final imputed results. We included variables from the feature importance in ML analysis as additional covariates for the complex models: smoking (reference=nonsmokers), the presence of diabetes (reference=absence), age, BMI, creatinine, HDLc, LDLc, triacylglycerols, CRP, hemoglobin, SBP, and DBP. All continuous variables (except CC-IMT) were mean-centered for the analyses [32].

#### Subgroup analysis

We conducted a subgroup analysis for children and adolescents (age< 18y); here, we also used spline regression (degree of freedom = 4) and used sex as an interaction with iron biomarker (as above). Random-effect models were applied for iron and ferritin analyses in this age group. However, for the analyses of transferrin and transferrin saturation (TSAT) in children and adolescents, study could not be included as a random effect because only one study reported data on these parameters. Therefore, here we fitted using linear regression (without including study as a random effect).

Further, we also conducted subgroup analyses among adults to test the if the associations for ferritin and transferrin from the main analysis also held true for specific populations such as nonsmokers and normotension. For this, the multivariable regression models were built like for the complex models (as described above).

### UK Biobank

Further analyses were performed in the UK Biobank to determine the associations between hemochromatosis (HFE)-genotype groups and CC-IMT. UK Biobank includes ~ 500,000 community volunteers aged 39–73 years at baseline assessment (2006–2010) from 22 assessment centers across England, Scotland and Wales [as described elsewhere [33, 34] ]. We included participants genetically similar to the 1000 Genomes project European reference population [35] [the categorization of this population is described elsewhere [36] ], with HFE p.C282Y (rs1800562) and HFE p.H63D (rs1799945) genotype data from whole exome sequencing [methods developed by Regeneron [37] ]. We analyzed a subset of these participants with available carotid ultrasound data from an imaging visit starting in 2014 [*n* = 42,299; [38] ]. Four CC-IMT variables were available in the UK Biobank imaging study [at 120, 150, 210 and 240 degrees; variable IDs 22671, 22674, 22677, 22680 [38] ]; from these, we calculated an overall mean CC-IMT value and performed linear regression analyses to test associations with C282Y/H63D genotype groups [C282Y^(−/−)^ H63D^(+/−)^; C282Y^(−/−)^ H63D^(+/+)^; C282Y^(+/−)^ H63D^(+/−)^; C282Y^(+/−)^ H63D^(−/−)^; C282Y^(+/+)^ H63D^(−/−)^], compared to those with no mutations [C282Y^(−/−)^H63D^(−/−)^]. Models were stratified by sex, and adjusted for age, assessment centre and ten genetic principal components (to account for genetic stratification). We also performed sensitivity analyses by repeating the analysis (as above) after excluding participants diagnosed with hemochromatosis.

## Results

### Process of data collection

We identified a total of 1,032 records via literature search, of which 887 were excluded by screening the title and abstract. The full text of the remaining 145 articles was investigated, and 108 publications were selected for the meta-analysis. We contacted the authors of the selected publications, from which we received IPD data from 22 studies (IPD retrieval 20.4%) [39–59]. Of these, we excluded two studies that did not clear the data integrity checks [58, 59]. Additionally, we included our own data from the HEIST-DiC study [11]. The flow of the literature search is shown in Fig. 2a and the ML pipeline is shown in Fig. 2b.

### Study characteristics

Eighteen studies included in the IPD were hospital-based [11, 39, 40, 42–50, 52–56, 60] and 3 studies were population-based [41, 51, 57]. Controls were part of the study population in 12 studies [39, 40, 42–44, 48–50, 53–55, 57]. The characteristics of studies and variables included in the IPD-MA are shown in Table 2 and Supplementary Table 2.

**Table 2:** Characteristics of Adult participants

Characteristic	Missing	Overall *N* = 7,523^1^	Males *N* = 4,974^1^	Females *N* = 2,549^1^	*p*-value^2^
Age (years)	0 (0%)	57 (47, 65)	55 (46, 64)	58 (50, 66)	< 0.001
BMI (kg/sq.m)	152 (2.0%)	27.2 (24.6, 30.2)	27.5 (25.2, 30.2)	26.4 (23.4, 30.3)	< 0.001
Smoking					
Nonsmokers	328 (4.4%)	3,972 (52.8%)	2,600 (34.6%)	1,372 (18.2%)	0.009^c^
Former Smokers		1,912 (25.4%)	1,313 (17.5%)	599 (7.96%)	
Current Smokers		1,311 (17.4%)	907 (12.1%)	404 (5.4%)	
Diabetes					
No Diabetes	901 (12%)	5,480 (72.8%)	3,678 (48.9%)	1,802 (24%)	> 0.9^c^
Diabetes		1,142 (15.2%)	767 (10.2%)	375 (4.98%)	
Hypertension					
No hypertension	2,322 (31%)	1,717 (22.8%)	837 (11.1%)	880 (11.7%)	< 0.001^c^
Hypertension		3,484 (46.3%)	2,150 (28.6%)	1,334 (17.7%)	
SBP (mm/Hg)	310 (4.1%)	132 (120, 145)	132 (121, 145)	130 (118, 145)	< 0.001
DBP (mm/Hg)	310 (4.1%)	80 (73, 88)	80 (75, 88)	78 (70, 86)	< 0.001
CKD					
No CKD	2,490 (33%)	2,467 (32.8%)	1,999 (26.6%)	468 (6.2%)	< 0.001^c^
CKD		2,566 (34.1%)	1,606 (21.3%)	960 (12.8%)	
Anemia					
No Anemia	3,533 (47%)	2,737 (36.4%)	1,573 (20.9%)	1,164 (15.5%)	< 0.001^c^
Anemia		1,253 (16.7%)	804 (10.7%)	449 (5.96%)	
Hemoglobin (g/dL)	3,532 (47%)	13.40 (12.10, 14.70)	14.00 (12.40, 15.20)	12.80 (11.90, 13.80)	< 0.001
Creatinine (mg/dL)	3,156 (42%)	1.05 (0.86, 1.83)	1.22 (0.96, 2.10)	0.89 (0.77, 1.49)	< 0.001
HDLc (mg/dL)	784 (10%)	49 (41, 59)	46 (39, 55)	57 (47, 68)	< 0.001
LDLc (mg/dL)	882 (12%)	118 (94, 145)	118 (94, 143)	120 (95, 148)	0.005
Triacylglycerols (mg/dL)	512 (6.8%)	124 (88, 180)	127 (89, 186)	119 (84, 170)	< 0.001
CRP (mg/dL)	5,476 (73%)	4.00 (1.30, 4.00)	4.00 (1.29, 4.00)	4.00 (1.40, 4.00)	0.14
CC-IMT (mm)	107 (1.4%)	0.73 (0.60, 0.84)	0.74 (0.60, 0.85)	0.71 (0.60, 0.82)	< 0.001
Iron (µM)	4,271 (57%)	17.0 (14.0, 21.0)	18.0 (14.0, 22.0)	16.0 (12.5, 19.9)	< 0.001
Ferritin (ng/mL)	1,786 (24%)	131 (66, 233)	156 (87, 260)	89 (45, 171)	< 0.001
Transferrin (g/L)	4,594 (61%)	231 (199, 263)	230 (199, 261)	233 (199, 269)	0.012
TSAT (%)	2,713 (36%)	28 (22, 35)	30 (23, 37)	26 (20, 32)	< 0.001

The demographics of the adult participants (age≥18y) stratified by sex are shown. For continuous variables, the ^1^summary data are shown as Median (IQR) while for categorical variables, the data are represented as N (%).^2^P-values as calculated by Wilcoxon rank sum test (for continuous variables) or ^c^Pearson’s Chi-Squared test (for categorical variables); significant P-values are highlighted in bold. Missing data are shown as N (%). All percentages shown are based on the total number of subjects

*Abbreviations BMI* Body Mass Index, *HDLc* High-Density Lipoprotein, *LDLc* Low-Density Lipoprotein, *CRP* C-reactive protein, *CKD* Chronic Kidney Disease, *CC-IMT* Carotid Intima-Media Thickness, *TSAT* Transferrin Saturation, *SBP* Systolic Blood Pressure, *DBP* Diastolic Blood Pressure

### Participant characteristics

The pooling of all available datasets yielded a total of 10,807 participants. From these, we excluded participants with CC-IMT value indicative of atherosclerotic plaque [20] (> 1.5 mm; *N* = 47), CRP indicative of overt inflammation (> 10 mg/dL; *N* = 162) and all conditions suggestive of an elevated iron status [possible hemochromatosis [21] (*N* = 178), previously diagnosed thalassemia (*N* = 131) or hemochromatosis (*N* = 74)]. This resulted in a final pooled participant size of 10,215. Most of the participants were adults except for 4 studies [47, 49, 55, 57] which had included children and adolescents in their work (age< 18y). Eighteen studies [11, 39–42, 46–57] included male and female participants, while 3 studies [43–45] included only males in their study population. A study-wise breakdown of participant characteristics is shown in Supplementary Table 3.

#### Adults

The demographics of the adult participants (*N* = 7,523) are shown in Table 1. Here, the proportion of males (*N* = 4,974; 66.12%) was higher than females (*N* = 2,549; 33.88%). Overall, the males were younger, had a higher BMI and a greater proportion of smokers (*p* = 0.009). The proportion of participants with hypertension (*p* < 0.0001) was also higher among males; in line with this observation, the systolic blood pressure (SBP) and diastolic blood pressure (DBP) of males were higher (Table 1). On the other hand, more female participants in the cohort had CKD (44.55%; *p* < 0.0001; Table 1). Serum iron, ferritin, and TSAT were higher, while transferrin levels were lower among males than females (Table 1; Supplementary Figs. 2–5).

#### Children and adolescents

Among children and adolescents, age (*p* = 0.131) and BMI (*p* = 0.838) of the participants among both sexes were not different (Table 3). The proportion of smokers was higher among females than males (76% vs. 24%; *p* = 0.002). Overall, females had lower hemoglobin levels (*p* < 0.0001). CRP levels were not different between the sexes (*p* = 0.226). Data on the presence of comorbidities were not available for most of the participants (> 98%; Table 3). With regards to the iron parameters, males had higher ferritin and transferrin levels while iron and TSAT levels were not different between the sexes (Table 3; Supplementary Figs. 2–5).

**Table 3:** Characteristics of children and adolescents

**Characteristic**	**Missing**	**Overall**	**Males**	**Females**	p-value^2^
		N = 2,691^1^	N = 1,241^1^	N = 1,450^1^	
Age (years)	0 (0%)	15.5 (10.2, 16.5)	15.4 (9.7, 16.4)	15.6 (10.8, 16.5)	0.13
BMI (kg/sq.m)	12 (0.4%)	20.7 (18.4, 23.3)	20.8 (18.1, 23.4)	20.6 (18.5, 23.1)	0.8
SBP (mm/Hg)	18 (0.7%)	117 (108, 127)	121 (109, 131)	115 (107, 123)	<0.001
DBP (mm/Hg)	18 (0.7%)	68 (62, 74)	67 (61, 73)	69 (63, 75)	<0.001
Hemoglobin (g/dL)	70 (2.6%)	13.90 (13.00, 14.90)	14.70 (13.30, 15.70)	13.60 (12.90, 14.20)	<0.001
Creatinine (mg/dL)	9 (0.3%)	0.75 (0.57, 0.87)	0.81 (0.55, 0.93)	0.72 (0.59, 0.81)	<0.001
HDLc (mg/dL)	7 (0.3%)	56 (48, 66)	54 (47, 62)	59 (51, 69)	<0.001
LDLc (mg/dL)	7 (0.3%)	91 (77, 108)	88 (73, 105)	94 (80, 111)	<0.001
Triacylglycerols (mg/dL)	10 (0.4%)	65 (50, 89)	64 (47, 86)	67 (52, 92)	<0.001
CRP (mg/dL)	34 (1.3%)	0.09 (0.06, 0.40)	0.10 (0.06, 0.40)	0.09 (0.06, 0.36)	0.2
CC-IMT (mm)	132 (4.9%)	0.40 (0.36, 0.44)	0.41 (0.37, 0.45)	0.39 (0.35, 0.43)	<0.001
Iron (µM)	2,371 (88%)	12 (9, 16)	13 (9, 17)	12 (9, 16)	0.4
Ferritin (ng/mL)	21 (0.8%)	38 (24, 59)	47 (30, 73)	32 (19, 50)	<0.001
Transferrin (g/L)	1,885 (70%)	273 (251, 298)	277 (253, 303)	270 (249, 294)	0.041
TSAT (%)	2,394 (89%)	20 (15, 26)	20 (15, 26)	20 (15, 26)	0.8

The demographics of the children and adolescent participants (age< 18y) stratified by sex are shown. For continuous variables, the 1summary data are shown as Median (IQR) while for categorical variables, the data are represented as N (%).2P-values as calculated by the Wilcoxon rank sum test (for continuous variables); significant P-values are highlighted in bold. Missing data are shown as N (%). All percentages shown are based on the total number of subjects

*Abbreviations BMI *Body Mass Index,* HDLc,* High-Density Lipoprotein, *LDLc* Low-Density Lipoprotein, *CRP* C-reactive protein, *CC-IMT* Carotid Intima-Media Thickness, *TSAT* Transferrin Saturation, *SBP* Systolic Blood Pressure, *DBP* Diastolic Blood Pressure

### CC-IMT

In all studies, the CC-IMT was measured using ultrasound, but there were differences in the methods used (Supplementary Table 4). Among the adults, the CC-IMT [median (IQR); mm] of males [0.74 (0.6–0.85)] was higher compared to females [0.71 (IQR 0.6–0.82); *p* < 0.0001; Supplementary Fig. 6]. This was also the case among children and adolescents, with higher CC-IMT among males [0.41 (0.37–0.44)] compared to females [0.39 (0.35–0.43); *p* < 0.0001; Supplementary Fig. 6].

### Missing data

We observed two types of missing values in the IPD data: (1) systematically missing variables were present across studies (since not all studies collected the variables uniformly) (2) sporadically missing values within each study. The proportion of missing values for each variable in the pooled data is shown in Supplementary Table 5. A study-wise breakdown of missing variables is shown in Supplementary Tables 6 and an age-categorized breakdown is shown in Supplementary Table 7. The pattern of co-occurrence of missing values across variables is shown in Supplementary Fig. 7.

### Machine learning

**Fig. 3:**
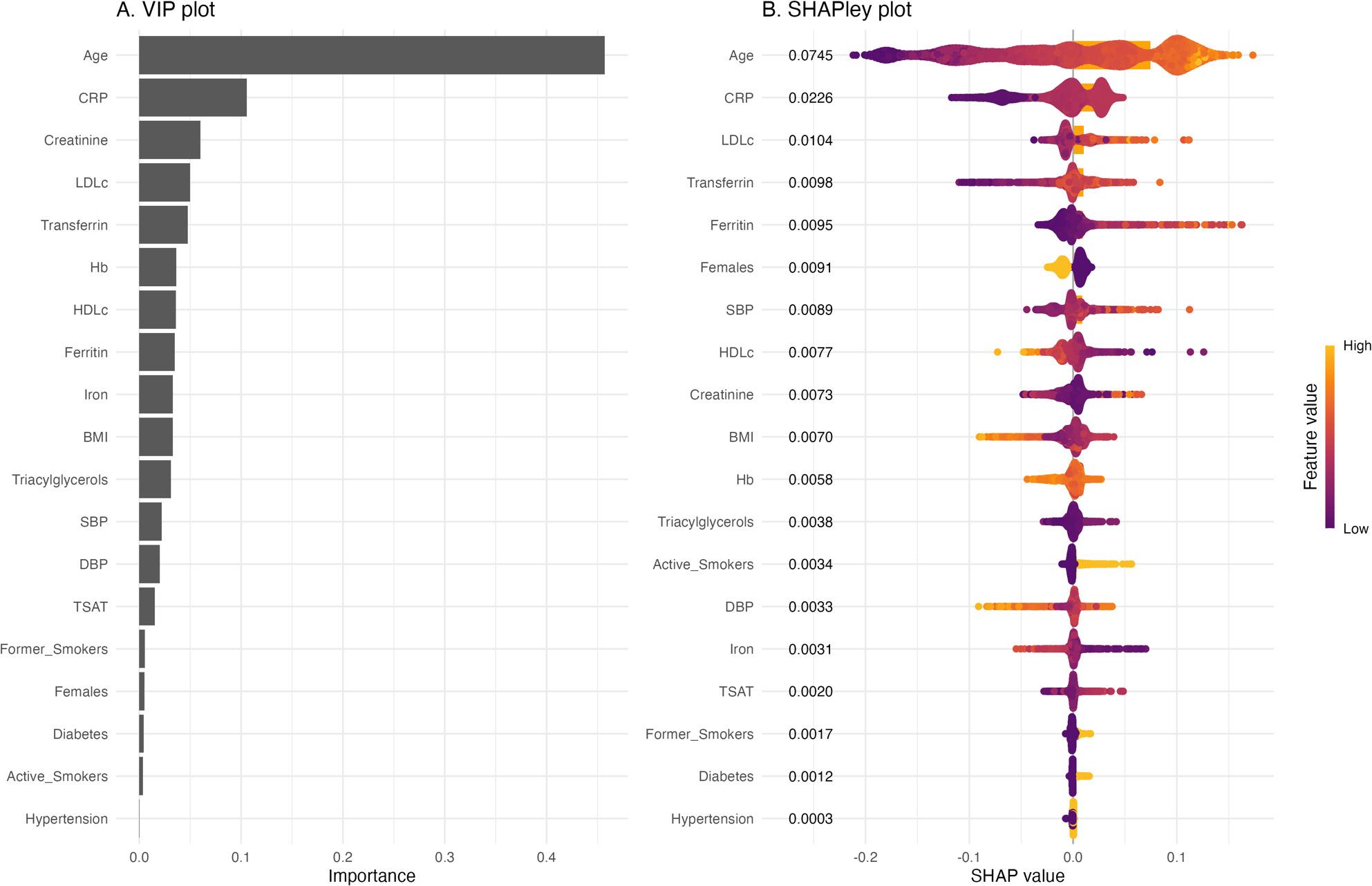
**a** Variables of importance identified in the machine learning prediction. The variable importance plot shows which predictors contribute most to the machine learning model's predictions of CC-IMT. They answer the question: "Which variables matter most to the model?". Variables are ranked from most to least important (top to bottom) based on their total contribution to model accuracy across all individuals. Bar length represents the average magnitude of each variable's influence on predictions (longer bars = greater overall importance). Only the top 15 predictors are displayed. **b** SHAPley plot of the variables contributing to the model performance. SHAP dependence plots show how each feature influences model predictions. In the SHAP dependence plots, each point represents one individual. They answer: "How does this variable affect different people?". The x-axis shows the feature value, and the y-axis shows the SHAP value (contribution to model prediction: positive = increases prediction, negative = decreases prediction, near zero = minimal contribution). Colour intensity reflects feature interaction strength

The dataset used for ML contained 5,740 participants and 21 covariates. The Training subset contained 4,306 (75%) participants while the Test subset contained 1,434 (25%) participants. The baseline characteristics between the two subsets were comparable (Supplementary Table 8). The ML model demonstrated moderate predictive performance with an R2 of 0.447, indicating that it explained 44.7% of the variance in the outcome variable; the Root Mean Square Error (RMSE) and the Mean Average Error (MAE) of the model were 0.136 and 0.101, respectively. The feature importance plot shows the contribution of each variable for the prediction of the overall model (Fig. 3a). On the other hand, the SHAP plot highlights how the variables and their individual values contribute to the prediction (Fig. 3b). Together, these plots emphasize the importance of each variable in our ML model’s predictions. All the iron biomarkers (transferrin, ferritin, TSAT and iron) were ranked among important predictors of CC-IMT. In addition, age, sex, CRP, creatinine, LDLc, HDLc, hemoglobin, systolic and diastolic BP, triacylglycerols, and smoking were also identified as important features.

### Regression analyses

#### Adults

In the analysis of unimputed data, we found that ferritin alone showed a positive effect within specific ranges [131–233 ng/mL: β = 0.08, 95% CI (0.002, 0.16), *p* = 0.046; >233 ng/mL: β = 0.16, 95% CI (0.04, 0.27), *p* = 0.008]. A significant interactive effect was also observed between females and ferritin with CC-IMT [ferritin > 233 ng/mL: β = 0.04, 95% CI (0.002, 0.08), *p* = 0.037; Supplementary Table 9]. The main effects of transferrin alone were all non-significant, however negative interactions were noted for transferrin within specific ranges among females tending to significance [231–263 mg/dL: β=-0.21, 95% CI (-0.43, 0.003), *p* = 0.054; >263 mg/dL: β=-0.73, 95% CI (-1.48, 0.01), *p* = 0.055; Supplementary Table 10]. On the other hand, none of the terms for iron or TSAT (including interactions with sex) showed statistically significant associations with CC-IMT (Supplementary Tables 11–12).

In the complex models (Tables 4 and 5), we confirmed potential sex-specific effects at higher levels of ferritin and transferrin. We found that ferritin alone showed a significant effect at a specific range (131–233 ng/mL: β = 0.13, 95% CI [0.02, 0.24], *p* = 0.038). Further, we found a positive interaction between ferritin with CC-IMT among females with CC-IMT specifically at the higher ranges (> 233 ng/mL: β = 0.54, 95% CI [0.11, 0.97], *p* = 0.015). Although transferrin alone showed no significant effects with CC-IMT, we found significant interactions among females at a specific range (231–263 mg/dL: β= -0.99, 95% CI [-1.81, -0.17], *p* = 0.039). The effect of sex alone was nonsignificant in both models. Other significant variables were age (β = 0.14, *p* < 0.001), diabetes (β = 0.02, *p* < 0.001), HDL-C (β = -0.006, *p* = 0.003), LDL-C (β = 0.008, *p* < 0.001), smoking (β = 0.016, *p* < 0.001 for former smokers; β = 0.019, *p* < 0.001 for current smokers), CRP (β = 0.01, *p* = 0.024), hemoglobin (β = 0.0098, *p* = 0.010), systolic blood pressure (β = 0.018, *p* < 0.001), and diastolic blood pressure (β = -0.0087, *p* = 0.0006); all of these significantly associated with CC-IMT (Tables 4 and 5).

**Table 4:** Multivariable regression analysis of ferritin and CC-IMT

Parameter	Estimate	95% CI		Standard Error	Fraction of Missing Information (FMI)	Relative Increase in Variance (RIV)	p-value
		Low	High				
Intercept	0.617	0.464	0.77	0.078	0.485	0.779	< 0.0001
Ferritin (ng/mL)							
< 66.4	0.025	-0.113	0.163	0.07	0.548	0.983	0.726
66.4-130.5	0.1303	0.0192	0.241	0.057	0.591	1.156	0.0376
130.6-232.9	0.141	-0.168	0.45	0.158	0.559	1.023	0.386
> 233	0.082	-0.102	0.265	0.094	0.174	0.195	0.383
Sex: F (ref = M)	-0.002	-0.175	0.171	0.089	0.103	0.11	0.982
Ferritin (ng/mL): Females							
< 66.4	-0.025	-0.195	0.146	0.087	0.094	0.0995	0.774
66.4-130.5	-0.078	-0.243	0.087	0.084	0.343	0.453	0.358
130.6-232.9	0.251	-0.153	0.655	0.206	0.127	0.139	0.224
> 233	0.54	0.107	0.974	0.221	0.07	0.073	0.0148
Other covariates							
Age	0.139	0.132	0.146	0.003	0.046	0.047	< 0.0001
Diabetes (ref = No Diabetes)	0.025	0.014	0.036	0.006	0.436	0.65	0.0001
Creatinine	-0.006	-0.016	0.003	0.005	0.764	2.458	0.215
HDLc	-0.006	-0.01	-0.002	0.002	0.268	0.326	0.0035
LDLc	0.008	0.0043	0.012	0.0020	0.3757	0.516	0.0003
Triacylglycerols	-0.003	-0.006	0.008	0.002	0.077	0.08	0.1324
BMI	0.004	-0.0002	0.008	0.002	0.044	0.045	0.066
Former smokers (ref=Nonsmokers)	0.017	0.008	0.025	0.0042	0.047	0.048	0.0001
Active smokers (ref=Nonsmokers)	0.019	0.0102	0.029	0.0047	0.243	0.288	0.0001
CRP	0.01	0.004	0.017	0.004	0.84	3.78	0.024
Hemoglobin	0.0098	0.004	0.016	0.0032	0.66	1.51	0.0103
SBP	0.018	0.013	0.023	0.0035	0.213	0.247	< 0.0001
DBP	-0.009	-0.014	-0.004	0.0035	0.195	0.23	0.0006

We flexibly modelled the relationship between ferritin and CC-IMT using spline regression (degree of freedom=4). In addition, we tested the associations between ferritin and CC-IMT for differences due to sex- by using an interaction term (ferritin*sex; Males as reference). All continuous variables were mean-centered for the analyses

*Abbreviations BMI *Body Mass Index, *HDLc* High-Density Lipoprotein, *LDLc* Low-Density Lipoprotein, *CRP* C-reactive protein, *CC-IMT* Carotid Intima-Media Thickness, *SBP* Systolic Blood Pressure, *DBP* Diastolic Blood Pressure

**Table 5:** Multivariable regression analysis of transferrin and CC-IMT

Parameter	Estimate	95% CI		Standard Error	Fraction of Missing Information (FMI)	Relative Increase in Variance (RIV)	*p*-value
		Low	High				
Intercept	0.691	0.557	0.824	0.068	0.586	1.133	< 0.0001
Transferrin (g/L)							
< 199	-0.052	-0.176	0.073	0.064	0.755	2.35	0.44
199–230	-0.0116	-0.0834	0.06	0.037	0.507	0.85	0.76
231–263	-0.1428	-0.4088	0.12	0.136	0.701	1.818	0.318
> 263	-0.181	-0.379	0.007	0.098	0.259	0.313	0.063
Sex: F (ref = M)	0.489	0.057	0.921	0.22	0.74	2.14	0.055
Transferrin (g/L): Females							
< 199	-0.4991	-0.9282	-0.0700	0.2189	0.7429	2.2095	0.0504
199–230	-0.3030	-0.5645	-0.0415	0.1334	0.7488	2.2752	0.0517
231–263	-0.9900	-1.8072	-0.1728	0.4169	0.6903	1.7307	0.0391
> 263	-0.1173	-0.4455	0.2108	0.1674	0.4959	0.8109	0.4916
Other covariates							
Age	0.139	0.132	0.146	0.0034	0.039	0.04	< 0.0001
Diabetes (ref = No Diabetes)	0.025	0.015	0.036	0.006	0.419	0.609	0.0001
Creatinine	-0.006	-0.015	0.003	0.005	0.758	2.39	0.227
HDLc	-0.006	-0.01	-0.002	0.002	0.254	0.305	0.005
LDLc	0.009	0.005	0.013	0.002	0.346	0.458	0.0001
Triacylglycerols	-0.002	-0.005	0.002	0.002	0.09	0.093	0.3
BMI	0.004	-0.0001	0.008	0.002	0.113	0.12	0.058
Former smokers (ref=Nonsmokers)	0.017	0.008	0.025	0.004	0.069	0.072	0.0001
Active smokers (ref=Nonsmokers)	0.019	0.01	0.029	0.005	0.23	0.28	0.0001
CRP	0.012	0.006	0.018	0.003	0.804	3.09	0.008
Hemoglobin	0.01	0.004	0.017	0.003	0.69	1.74	0.0128
SBP	0.018	0.013	0.023	0.003	0.22	0.26	< 0.0001
DBP	-0.009	-0.013	-0.004	0.002	0.153	0.17	0.0006

We flexibly modelled the relationship between transferrin and CC-IMT using spline regression (degree of freedom=4). In addition, we tested the associations between transferrin and CC-IMT for differences due to sex- by using an interaction term (transferrin *sex; Males as reference). All continuous variables were mean-centered for the analyses

*Abbreviations BMI* Body Mass Index, *HDLc* High-Density Lipoprotein, *LDLc* Low-Density Lipoprotein, *CRP* C-reactive protein, *CC-IMT* Carotid Intima-Media Thickness, *SBP* Systolic Blood Pressure, *DBP* Diastolic Blood Pressure

#### Subgroup analysis

##### Children and adolescents

We found no significant associations between any of the iron biomarkers and CC-IMT among children and adolescents and no notable interactions with sex (Supplementary Tables 13–16).

##### Nonsmokers

In contrast, no significant associations were observed between ferritin or transferrin and CC-IMT among nonsmokers (Supplementary Tables 17–18).

##### Normotensive individuals

Among normotensive individuals, we found that spline regression showed significant interactions for ferritin and transferrin with sex, confirming the non-linear and female-specific associations from the main analysis (Supplementary Tables 19–20). In females, ferritin > 232 ng/mL were associated with higher CC-IMT (β = 1.22; 95% CI 0.37–2.07; *p* = 0.005). In this subgroup, the associations for transferrin were also significant among females (< 196 mg/dL: β = −0.62, 95% CI [− 1.03, − 0.2], *p* = 0.007; 197–231 mg/dL: β = −0.34, 95% CI [− 0.62, − 0.06]; *p* = 0.029; 232–259 mg/dL: β = −1.23 [95% CI − 2.1 to − 0.37; *p* = 0.009)

### UK Biobank

Further, we analysed data from 42,299 UK Biobank participants for associations between HFE-genotypes and mean CC-IMT. Here, no statistically significant associations were detected in either males or females between CC-IMT in those with C282Y/H63D genotypes (including C282Y homozygotes) compared to those with no mutations (Supplementary Table 21). The associations remained non-significant in a sensitivity analysis, restricted to participants who were undiagnosed with hemochromatosis (*N* = 42,193).

## Discussion

To investigate the conflicting conclusions of the relationship between iron biomarkers and CC-IMT in previous studies, we conducted and report the first IPD meta-analysis. This meta-analysis includes diversely distributed studies (18 hospital-based and 3 population-based studies) and a study population comprising both adults (*N* = 7,523) and children/adolescents (*N* = 2,691). Although males were over-represented in the study population, the proportion of females in both age groups were sufficient to analyze sex-specific differences. The study participants showed different comorbidities e.g., diabetes, hypertension, and CKD. Therefore, this comprehensive dataset, the largest to date, allows robust exploration to test associations between iron parameters and CC-IMT across age groups and clinical settings.

Our observations from the multivariable models also align with established biological mechanisms underlying vascular remodeling and atherosclerosis. Diabetes showed a positive association with CC-IMT, consistent with the well-documented impact of chronic hyperglycemia on endothelial dysfunction. Consistently, the effect of hypertension was also affirmed by the positive association between SBP and CC-IMT. Similarly, higher LDL-cholesterol and lower HDL-cholesterol were associated with increased CC-IMT, reflecting their respective roles in atherogenesis. Elevated CRP was positively associated with CC-IMT, supporting the role of inflammation. Both former and current smoking were associated with higher CC-IMT relative to nonsmokers, underscoring the persistent vascular effects of tobacco exposure, even after cessation. The small positive trend observed with BMI is also consistent with the known consequences of adiposity. Together, these results reinforce the biological plausibility of our models. Overall, the results suggest that elevated ferritin and reduced transferrin levels within the reference limits are associated with CC-IMT, particularly in adult females.

Our findings can be interpreted from an ‘iron perspective’: ferritin stores cellular iron and is a well-established marker of body iron stores [61]; transferrin carries iron in the blood and supplies all cell types with the metal. The observed association between elevated ferritin levels and CC-IMT could be interpreted as supportive of the “iron hypothesis” in atherosclerosis, which suggests that elevated iron stores contribute to oxidative stress and vascular damage [62, 63]. This idea is further supported by our observation that elevated transferrin levels (frequently observed in iron deficiency) are inversely associated with CC-IMT. A recent mendelian randomization study showing that an elevated iron status increases the risk of cardiovascular disease, specifically ischemic stroke provides additional support to this model [64]. In this context, a genome-wide association study by Galesloot et al. [65] showed that polymorphisms predicting higher hepcidin/ferritin ratios were associated with an increased atherosclerosis risk. Unfortunately, data on hepcidin and non-transferrin-bound iron (NTBI) were not available in our study to evaluate their associations, and we suggest that future studies may consider including additional biomarkers such as NTBI and hepcidin in their analysis.

As an extension of this idea, the logical argument would be that individuals with genetic iron overload could be at a higher risk of developing cardiovascular disease (CVD). We have previously shown that patients with genetic iron overload conditions (e.g., hemochromatosis or thalassemia major) show elevated markers of vascular dysfunction [Intercellular Adhesion Molecule 1 (ICAM-1), Vascular Adhesion Molecule 1 (VCAM-1)] that correlated positively with NTBI in these patients [1, 2]. Importantly, phlebotomy treatment of the hemochromatosis patients reverted the increased concentrations of ICAM-1 and VCAM-1 [1]. To follow up on these data, we also investigated the UK Biobank for associations between mean CC-IMT and the hemochromatosis genotype (C282Y/H63D genotypes) using MR, but we did not detect significant associations. MR offers an important complement to observational analyses by reducing bias from confounding and reverse causation. The contrasting absence of an MR association do not support a causal effect of genetically determined iron overload on CC-IMT and further suggest that our observational findings may reflect residual confounding, reverse causation, or other non-causal mechanisms.

Inflammation could be one such confounder since iron metabolism and inflammation are tightly interconnected processes. Therefore, our findings can also be interpreted from an ‘inflammation perspective’. Inflammation is known to aggravate atherosclerosis [66]. Ferritin is a well-known acute phase protein, which is induced in response to inflammation. Similarly, transferrin levels are reduced in inflammatory conditions [67]. Although, we have excluded individuals with CRP > 10 mg/dL in our analysis, we cannot completely exclude low-grade inflammation as a driver of increased CC-IMT. Thus, the opposing associations of ferritin and transferrin with CC-IMT could be an epiphenomenon to inflammation.

Biological sex is another factor that can influence iron metabolism and CC-IMT. It is well established that biological sex influences the risk for CVD [68]. Our recent work also detected a positive nonlinear association between ferritin and peripheral arterial disease in certain ferritin ranges specifically in females [48–97 ng/mL: OR 14.59, 95% CI 1.6–135.93, P= 0.019; 98–169 ng/mL: OR 171.07, 95% CI 1.27–23404, P= 0.039; [11]. Although the overall prevalence of CVD is higher among males, several studies show a higher risk of mortality and morbidity in females due to CVD, particularly in the presence of common risk factors and comorbidities [69, 70]. Female-specific risk factors include hormones, pregnancy and reproductive health (e.g. menstruation, pregnancy-associated disorders etc.). While the influence of female sexual hormones on iron homeostasis is known [9, 10], previous studies have not found consistent evidence linking factors such as parity, timing of menopause, duration of the reproductive period, use of hormone therapy or contraceptives with CC-IMT [71, 72]. We have accounted differences due to sex by using it as an interaction term and show that ferritin and transferrin show nonlinear associations specifically among females. Nevertheless, we were unable to adjust for effects of hormonal status or menstruation status in this study due to the unavailability of data. Further, whether these reproductive hormones also affect the production of other liver-expressed proteins, such as ferritin or transferrin is unclear.

The use of medications is another confounder affecting the interpretation of our study. Medications can affect CC-IMT progression and, in some cases, iron metabolism. For example, the use of statins has been associated with a significant reduction in CC-IMT progression [14, 73]. as well as lower ferritin levels [74, 75]. Additionally, other commonly used drugs such as metformin, glucagon like peptide-1 receptor agonists, dipeptidylpeptidase-4 inhibitors, phosphodiesterase III inhibitors, calcium channel blockers, and antiplatelet agents also attenuate CC-IMT progression [76–78], while the effects on iron metabolism remain incompletely understood. The datasets analyzed here lacked detailed information on medication, representing a limitation of our study.

Further, in our ML model, active smoking showed relatively lower importance (Fig. 3a-b) compared to other predictors. Part of the explanation for this is that ML approaches often assign lower importance to categorical factors with limited variability, whereas continuous variables such as BMI tend to carry greater predictive weight due to their wider distribution. Despite its lower importance in our context, smoking remains a well-established clinical risk factor and should not be interpreted as unimportant from a clinical perspective.

The strengths of our study include the comprehensive analysis of iron biomarkers with CC-IMT, thereby providing a rather complete picture of the role of systemic iron status. An additional strength of this meta-analysis is that it has achieved a good participant mix with studies from different geographical locations, a range of age groups (adults and children and adolescents), and common comorbidities such as CKD, diabetes, and hypertension.

The following limitations should however be considered when interpreting our findings. First, data retrieval for the IPD was incomplete, as we received only 20.4% of the published data despite our best efforts. We also had a significant proportion of missing data, both systematically across studies and sporadically within each study. Furthermore, it is essential to recognize that the data availability for these variables was not uniform across all studies; for example, we have not adjusted the models for the different treatments (e.g., medications for diabetes, lipid-lowering, dialysis for CKD, phlebotomy for hemochromatosis etc.) which could affect the CC-IMT outcome. It is worth noting that the data on additional iron biomarkers (hepcidin, NTBI) or menstrual and hormonal status were not available. In addition, the study population also does not represent all ethnicities, so we have not estimated effects due to these. While the inclusion of diverse cohorts enhances the generalizability of our findings, the heterogeneity in study designs and measurement protocols should be considered when interpreting the results. Despite the use of study-level random effects to account for between-study variability, residual confounding from unmeasured factors—such as differences in treatment regimens, comorbidities, and subtle variations in CC-IMT imaging protocols—cannot be excluded and may partly account for the observed associations. Therefore, the effect sizes observed in this study should be interpreted in the context of the known measurement variability of CC-IMT, including interobserver differences inherent to sonographic assessment [79].

Further, it is important to distinguish between statistical significance, biological plausibility, and clinical relevance. Statistical significance reflects the strength of evidence against the null hypothesis but may not necessarily imply a biologically plausible mechanism [80]. Importantly, even statistically significant and biologically plausible associations may not translate into clinically meaningful effects at the individual or population level [81]. Therefore, while our findings demonstrate statistical associations between certain iron biomarkers and CC-IMT in females, their clinical relevance at the individual level remains uncertain. In addition, the interpretation of iron metabolism biomarkers, particularly ferritin, is complicated by significant variability in assay standardization and traceability [82], representing another limitation of our study. This variability hinders the establishment of universal reference intervals or thresholds (from the spline knots) and complicates their clinical interpretation. Therefore, the thresholds identified in our study should be considered exploratory and are not intended to serve as cut-offs for decision making. Because the investigated biomarkers are biologically related and not statistically independent, and the analyses were hypothesis-driven and prespecified, formal multiple testing correction was not applied. Nevertheless, the potential for type I error cannot be excluded. Additionally, our study is cross-sectional and does not assess the temporal relationship between the parameters and therefore, cannot imply causality. We also recognize that the integration of ML and conventional statistics is still at an early stage; nevertheless, exploring this interface is meaningful, especially for hypothesis generation. Finally, since the study relied on published data, an element of publication bias cannot be excluded, as studies with non-significant results may remain unpublished and therefore, undiscovered.

**Table:** 

**Box 1: Glossary of key machine learning terms used in the study**	
Term	Plain language explanation
Bagged tree imputation	Uses many simple decision trees to predict missing values based on patterns in complete data
Centered and scaled	Adjusts numeric variables to have mean = 0 and standard deviation = 1 for fair model comparisons
Gradient-boosted tree [XGBoost]	Builds hundreds of simple prediction trees sequentially; each new tree corrects errors from previous ones
Hyperparameter tuning	Systematically tests different model settings to find the combination that performs best
Grid search	Tries all combinations from a predefined set of model settings
10-fold cross-validation	Splits data into 10 parts, trains on 9 parts and tests on 1 part (repeated 10 times) to get reliable performance estimates
Root Mean Squared Error (RMSE)	Average prediction error (lower = better model)
Variable importance (VIP)	Shows which predictors contribute most to model predictions overall
SHAP values	Quantifies how much each variable changes the prediction for each individual

## Conclusion

Our observational results demonstrate that iron biomarkers (specifically ferritin and transferrin) are non-linearly associated with CC-IMT, specifically in females. However, a significant causal association between HFE genotypes and CC-IMT in the UK Biobank data were not detected. This discrepancy may indicate that the observational associations reflect residual confounding (such as inflammation, medications), rather than a direct causal effect of iron status on arterial wall thickness. Future studies may want to consider these confounding factors alongside iron status indicators to better disentangle their specific contributions to atherosclerosis. We consider our findings exploratory to drive further research addressing the underlying mechanisms that could explain these associations.

## Ethics statement

All primary studies were approved by the local Ethics committees. All participants were included in the study according to the guidelines of the local ethics committees following written informed consent to participate. The North West Multi-Centre Research Ethics Committee (Research Ethics Committee reference 11/NW/0382) approved UK Biobank, and all participants provided their written informed consent at baseline. All research was conducted in accordance with both the Declarations of Helsinki and Istanbul.

## Supplementary Information

Supplementary Material 1: Supplementary Tables 1,2,4, 8-20

Supplementary Material 2: Contains Supplementary Figures 1-7

Supplementary Material 3: Study protocol as published in the PROSPERO database

Supplementary Material 4: PRISMA-IPD Checklist

Supplementary Material 5: Table of the studywise breakdown of participant characteristics

Supplementary Material 6: Table of the breakdown of the proportion of missing values for each variable in the pooled data

Supplementary Material 7: Table of the studywise breakdown of the proportion of missing values for each variable

Supplementary Material 8: Table of the breakdown of the proportion of missing values for each variable categorised by age group

Supplementary Material 9: Pseudocode of the Machine learning pipeline used for IMT prediction

Supplementary Material 10: Associations between Hemochromatosis HFE-genotypes and mean CC-IMT in the UK Biobank data

## Data Availability

Part of the data (subject to data sharing restrictions) may be available from authors upon reasonable request. The pseudocode for machine learning pipeline is provided in Supplementary File 3. Codes used for data analysis and visualization are accessible here: [https://github.com/griffindoc/imt](https:/github.com/griffindoc/imt).
