# Supplementary material for: Ferritin and transferrin predict common carotid intima-media thickness in females: a machine-learning informed individual participant data meta-analysis
Source: BMC Cardiovasc Disord. 2026 Apr 14;26:360. doi: 10.1186/s12872-026-05796-8 (PMC13123155; doi:10.1186/s12872-026-05796-8)
Supplement: Supplementary file 1 — Supplementary Material 1: Supplementary Tables 1,2,4, 8-20 [file 12872_2026_5796_MOESM1_ESM.docx]

Supplementary files: Association of iron indices with carotid intima-media thickness as a measure of atherosclerosis: an individual participant data meta-analysis

#

| Supplementary Table 1: R packages used for analysis   \| Package \| Version \| Citation \| \| --- \| --- \| --- \| \| base \| 4.3.1 \| ^1^ \| \| beepr \| 1.3 \| ^2^ \| \| broomExtra \| 5.0.0 \| ^3^ \| \| classInt \| 0.4.10 \| ^4^ \| \| docxtractr \| 0.6.5 \| ^5^ \| \| DT \| 0.30 \| ^6^ \| \| easystats \| 0.6.0 \| ^7^ \| \| flexdashboard \| 0.6.2 \| ^8^ \| \| flextable \| 0.9.4 \| ^9^ \| \| foreign \| 0.8.85 \| ^10^ \| \| formattable \| 0.2.1 \| ^11^ \| \| ggdist \| 3.3.0 \| ^12^ \| \| gghalves \| 0.1.4 \| ^13^ \| \| ggmice \| 0.1.0 \| ^14^ \| \| ggpackets \| 0.2.1 \| ^15^ \| \| ggplotify \| 0.1.2 \| ^16^ \| \| ggpubr \| 0.6.0 \| ^17^ \| \| ggtext \| 0.1.2 \| ^18^ \| \| gridExtra \| 2.3 \| ^19^ \| \| gt \| 0.10.0 \| ^20^ \| \| gtsummary \| 1.7.2 \| ^21^ \| \| here \| 1.0.1 \| ^22^ \| \| janitor \| 2.2.0 \| ^23^ \| \| jomo \| 2.7.6 \| ^24^ \| \| kableExtra \| 1.3.4 \| ^25^ \| \| knitr \| 1.45 \| ^26^ \| \| labelled \| 2.12.0 \| ^27^ \| \| lattice \| 0.22.5 \| ^28^ \| \| lme4 \| 1.1.35 \| ^29^ \| \| lmerTest \| 3.1.3 \| ^30^ \| \| mice \| 3.16.0 \| ^31^ \| \| mitml \| 0.4.5 \| ^32^ \| \| mitools \| 2.4 \| ^33^ \| \| naniar \| 1.0.0 \| ^34^ \| \| officer \| 0.6.3 \| ^35^ \| \| pacman \| 0.5.1 \| ^36^ \| \| pan \| 1.9 \| ^37^ \| \| RColorBrewer \| 1.1.3 \| ^38^ \| \| renv \| 1.0.3 \| ^39^ \| \| rmarkdown \| 2.25 \| ^40^ \| \| rsconnect \| 1.1.1 \| ^41^ \| \| rvg \| 0.3.3 \| ^42^ \| \| rworldmap \| 1.3.8 \| ^43^ \| \| shiny \| 1.7.5.1 \| ^44^ \| \| tidyverse \| 2.0.0 \| ^45^ \| \| writexl \| 1.4.2 \| ^46^ \| |
| --- | --- | --- | --- | --- | --- | --- | --- | --- | --- | --- | --- | --- | --- | --- | --- | --- | --- | --- | --- | --- | --- | --- | --- | --- | --- | --- | --- | --- | --- | --- | --- | --- | --- | --- | --- | --- | --- | --- | --- | --- | --- | --- | --- | --- | --- | --- | --- | --- | --- | --- | --- | --- | --- | --- | --- | --- | --- | --- | --- | --- | --- | --- | --- | --- | --- | --- | --- | --- | --- | --- | --- | --- | --- | --- | --- | --- | --- | --- | --- | --- | --- | --- | --- | --- | --- | --- | --- | --- | --- | --- | --- | --- | --- | --- | --- | --- | --- | --- | --- | --- | --- | --- | --- | --- | --- | --- | --- | --- | --- | --- | --- | --- | --- | --- | --- | --- | --- | --- | --- | --- | --- | --- | --- | --- | --- | --- | --- | --- | --- | --- | --- | --- | --- | --- | --- | --- | --- | --- | --- | --- | --- |

| Supplementary Table 2: Variables included in IPD-MA   \| **Variables** \| **Coding/Units** \| **Harmonization required** \| **N of participants** \| **N of studies** \| **IPD source** \| \| --- \| --- \| --- \| --- \| --- \| --- \| \| **Age** \| Years \| No \| 10214 \| 20 \| ^47–66^ \| \| **BMI** \| Kg/sq.m \| No \| 10050 \| 16 \| ^47–62^ \| \| **Sex** \| 0=males/ 1=females \| Yes \| 10214 \| 20 \| ^47–66^ \| \| **Smoking** \| 0=Nonsmoker/1=Former smoker/2=Current smoker \| Yes \| 8999 \| 14 \| ^47–49,52,53,55–58,60,61,63,64^ \| \| **Diabetes** \| 0=No/ 1=Yes \| Yes \| 6626 \| 14 \| ^48,51,53,55–61,63–66^ \| \| **Hypertension** \| 0=No/ 1=Yes \| Yes \| 5206 \| 13 \| ^51–53,55–61,64–66^ \| \| **CKD** \| 0=No/ 1=Yes \| Yes \| 5083 \| 8 \| ^47,48,50,52,53,61,65^ \| \| **Iron** \| µM \| Yes \| 3572 \| 16 \| ^47–52,54–59,61,62,64,65^ \| \| **Ferritin** \| ng/mL \| Yes \| 8407 \| 17 \| ^47–62,65^ \| \| **Transferrin** \| mg/dL \| Yes \| 3735 \| 10 \| ^49,51,53,54,56–59,64,65^ \| \| **TSAT** \| % \| No \| 5107 \| 9 \| ^47–49,52–55,59,61^ \| \| **Creatinine** \| mg/dL \| Yes \| 7049 \| 10 \| ^47,50,52–55,60–62^ \| \| **Triacylglycerols** \| mg/dL \| Yes \| 9692 \| 17 \| ^47–63^ \| \| **HDLc** \| mg/dL \| Yes \| 9423 \| 16 \| ^47–60,62,63^ \| \| **LDLc** \| mg/dL \| Yes \| 9325 \| 16 \| ^47–60,62,63^ \| \| **CRP** \| µg/dL \| Yes \| 4704 \| 11 \| ^50,52,54–61,63^ \| \| **Hemoglobin** \| g/dL \| No \| 6612 \| 13 \| ^47,51–54,56–63^ \| \| **SBP** \| mm Hg \| No \| 9886 \| 15 \| ^47–51,53–58,60,61,63^ \| \| **DBP** \| mm Hg \| No \| 9886 \| 15 \| ^47–51,53–58,60,61,63^ \| \| **Thalassemia** \| 0=No/ 1=Yes \| Yes \| 131 \| 3 \| ^63,66,67^ \| \| **Hemochromatosis** \| 0=No/ 1=Yes \| Yes \| 1276 \| 2 \| ^48,49^ \| |
| --- | --- | --- | --- | --- | --- | --- | --- | --- | --- | --- | --- | --- | --- | --- | --- | --- | --- | --- | --- | --- | --- | --- | --- | --- | --- | --- | --- | --- | --- | --- | --- | --- | --- | --- | --- | --- | --- | --- | --- | --- | --- | --- | --- | --- | --- | --- | --- | --- | --- | --- | --- | --- | --- | --- | --- | --- | --- | --- | --- | --- | --- | --- | --- | --- | --- | --- | --- | --- | --- | --- | --- | --- | --- | --- | --- | --- | --- | --- | --- | --- | --- | --- | --- | --- | --- | --- | --- | --- | --- | --- | --- | --- | --- | --- | --- | --- | --- | --- | --- | --- | --- | --- | --- | --- | --- | --- | --- | --- | --- | --- | --- | --- | --- | --- | --- | --- | --- | --- | --- | --- | --- | --- | --- | --- | --- | --- | --- | --- | --- | --- | --- | --- |

| Supplementary Table 4: Methodological differences in CC-IMT measurement   \| **Parameter** \| **Methodological details** \| **References** \| \| --- \| --- \| --- \| \| Distance proximal to the bifurcation where measurement was taken \| 1 cm \| ^48,50–52,55–59,61,66,67^ \| \|  \| 2 cm \| ^49^ \| \|  \| 2-3 cm \| ^47^ \| \|  \| Not specified \| ^53,60,63–65^ \| \| Number of measurements taken for calculation of mean CC-IMT \| 6 measurements \| ^62,63^ \| \|  \| 4 measurements \| ^51^ \| \|  \| 5 measurements \| ^54^ \| \|  \| 3 measurements \| ^60^ \| \|  \| Whole circumference \| ^50^ \| \|  \| Not specified \| ^47–49,52,53,55–59,61,64–67^ \| \| Frequency of transducer probe used \| 12 MHz \| ^54^ \| \|  \| 7.5 MHz \| ^50^ \| \|  \| 5-12 MHz \| ^63^ \| \|  \| Not specified \| ^47–49,51–53,55–62,64–67^ \| |
| --- | --- | --- | --- | --- | --- | --- | --- | --- | --- | --- | --- | --- | --- | --- | --- | --- | --- | --- | --- | --- | --- | --- | --- | --- | --- | --- | --- | --- | --- | --- | --- | --- | --- | --- | --- | --- | --- | --- | --- | --- | --- | --- | --- | --- | --- |

# 4. Supplementary Table 8: Comparison of baseline characteristics between the Training and Test subsets used for Machine Learning

| **Characteristic** | **Test subset**  **N = 1,436^1^** | **Training subset**  **N = 4,304^1^** | **p-value^2^** |
| --- | --- | --- | --- |
| **CC-IMT** | 0.73 (0.59, 0.84) | 0.72 (0.59, 0.84) | >0.9 |
| **Iron** | 17.0 (13.3, 21.0) | 17.2 (14.0, 21.0) | 0.093 |
| **Ferritin** | 127 (67, 235) | 130 (66, 232) | 0.8 |
| **Transferrin** | 230 (199, 264) | 232 (199, 263) | 0.7 |
| **TSAT** | 28 (21, 35) | 28 (22, 35) | 0.8 |
| **Ag** | 57 (45, 65) | 57 (45, 65) | 0.6 |
| **Creatinine** | 1.06 (0.85, 1.85) | 1.05 (0.86, 1.90) | 0.6 |
| **HDLc** | 50 (41, 61) | 49 (41, 60) | 0.5 |
| **LDLc** | 115 (91, 142) | 116 (92, 145) | 0.3 |
| **Triacylglycerols** | 122 (87, 181) | 127 (89, 181) | 0.2 |
| **BMI** | 26.8 (24.2, 29.8) | 26.9 (24.3, 30.0) | 0.2 |
| **CRP** | 4.00 (1.32, 4.00) | 4.00 (1.60, 4.00) | 0.6 |
| **Hb** | 13.20 (12.10, 14.50) | 13.20 (12.00, 14.50) | 0.4 |
| **SBP** | 132 (120, 145) | 132 (120, 145) | 0.8 |
| **DBP** | 80 (73, 88) | 80 (73, 87) | 0.8 |
| **Females** | 542 (38%) | 1,625 (38%) | >0.9 |
| **Diabetes** | 220 (18%) | 697 (19%) | 0.5 |
| **Hypertension** | 705 (67%) | 2,108 (67%) | 0.9 |
| **Former Smokers** | 371 (27%) | 1,156 (28%) | 0.6 |
| **Active Smokers** | 255 (19%) | 758 (19%) | 0.8 |
| ^1^Median (Q1, Q3); n (%) | | | |
| ^2^Wilcoxon rank sum test; Pearson's Chi-squared test | | | |

#

# Supplementary Table 9: Regression output Ferritin vs CC-IMT in adults

| **Parameter** | **Coefficient** | **Standard Error** | **95% CI** | | **T.Value** | **p-value** |
| --- | --- | --- | --- | --- | --- | --- |
|  |  |  | **Low** | **High** |  |  |
| **Intercept** | 0.675 | 0.043 | 0.589 | 0.760 | 15.513 | **<0.001** |
| **Ferritin (ng/mL)** | | | | | | |
| **<66.4** | 0.009 | 0.014 | -0.019 | 0.036 | 0.602 | 0.547 |
| **66.4-130.5** | -0.002 | 0.022 | -0.046 | 0.041 | -0.109 | 0.913 |
| **130.6-232.9** | 0.080 | 0.040 | 0.002 | 0.158 | 1.999 | **0.046** |
| **>233** | 0.158 | 0.059 | 0.041 | 0.274 | 2.653 | **0.008** |
| **Sex:F (ref=M)** | -0.072 | 0.018 | -0.107 | -0.037 | -4.032 | **<0.001** |
| **Ferritin (ng/mL): Females** | | | | | | |
| **<66.4** | 0.039 | 0.019 | 0.002 | 0.077 | 2.082 | **0.037** |
| **66.4-130.5** | 0.048 | 0.041 | -0.033 | 0.130 | 1.171 | 0.242 |
| **130.6-232.9** | 0.048 | 0.064 | -0.077 | 0.174 | 0.755 | 0.450 |
| **>233** | -0.053 | 0.121 | -0.290 | 0.185 | -0.433 | 0.665 |
| We fitted a linear mixed model (estimated using REML and nloptwrap optimizer) to predict CC-IMT with ferritin and sex (formula: imt ~ 1 + ns(ferritin, df = 4) * sex). The model included study as random effect (formula: ~1 \| study). CI = Confidence Interval | | | | | | |

# Supplementary Table 10: Regression output Transferrin vs CC-IMT in adults

| **Parameter** | **Coefficient** | **Standard Error** | **95% CI** | | **T.Value** | **p-value** |
| --- | --- | --- | --- | --- | --- | --- |
|  |  |  | **Low** | **High** |  |  |
| **Intercept** | 0.663 | 0.087 | 0.492 | 0.833 | 7.616 | **<0.001** |
| **Transferrin (g/L)** | | | | | | |
| **< 199** | 0.054 | 0.066 | -0.076 | 0.184 | 0.811 | 0.417 |
| **199-230** | 0.088 | 0.048 | -0.007 | 0.183 | 1.825 | 0.068 |
| **231-263** | 0.077 | 0.146 | -0.208 | 0.363 | 0.532 | 0.595 |
| **>263** | -0.084 | 0.132 | -0.342 | 0.175 | -0.635 | 0.526 |
| **Sex:F (ref=M)** | 0.305 | 0.185 | -0.058 | 0.667 | 1.647 | 0.100 |
| **Transferrin (g/L): Females** | | | | | | |
| **< 199** | -0.307 | 0.180 | -0.660 | 0.047 | -1.702 | 0.089 |
| **199-230** | -0.213 | 0.110 | -0.429 | 0.003 | -1.930 | 0.054 |
| **231-263** | -0.734 | 0.381 | -1.482 | 0.014 | -1.923 | 0.055 |
| **>263** | -0.068 | 0.176 | -0.412 | 0.276 | -0.389 | 0.698 |
| We fitted a linear mixed model (estimated using REML and nloptwrap optimizer) to predict CC-IMT with transferrin and sex (formula: imt ~ 1 + ns(transferrin, df = 4) * sex). The model included study as random effect (formula: ~1 \| study). CI = Confidence Interval | | | | | | |

#

# Supplementary Table 11: Regression output TSAT vs CC-IMT in adults

| **Parameter** | **Coefficient** | **Standard Error** | **95% CI** | | **T.Value** | **p-value** |
| --- | --- | --- | --- | --- | --- | --- |
|  |  |  | **Low** | **High** |  |  |
| **Intercept** | 0.741 | 0.049 | 0.646 | 0.837 | 15.209 | **<0.001** |
| **TSAT (%)** | | | | | | |
| **<21.8** | 0.019 | 0.032 | -0.043 | 0.082 | 0.605 | 0.545 |
| **21.8-28** | -0.057 | 0.032 | -0.120 | 0.006 | -1.775 | 0.076 |
| **28.1-35.1** | 0.004 | 0.086 | -0.165 | 0.173 | 0.048 | 0.962 |
| **>35.1** | -0.023 | 0.114 | -0.246 | 0.201 | -0.199 | 0.842 |
| **Sex:F (ref=M)** | -0.084 | 0.044 | -0.171 | 0.004 | -1.880 | 0.060 |
| **TSAT (%): Females** | | | | | | |
| **<21.8** | 0.048 | 0.043 | -0.036 | 0.131 | 1.115 | 0.265 |
| **21.8-28** | 0.045 | 0.068 | -0.088 | 0.178 | 0.666 | 0.505 |
| **28.1-35.1** | -0.058 | 0.233 | -0.514 | 0.399 | -0.247 | 0.805 |
| **>35.1** | -0.241 | 0.467 | -1.156 | 0.674 | -0.516 | 0.606 |
| We fitted a linear mixed model (estimated using REML and nloptwrap optimizer) to predict CC-IMT with TSAT and sex (formula: imt ~ 1 + ns(TSAT, df = 4) * sex). The model included study as random effect (formula: ~1 \| study). Complete (unimputed) data was used. CI = Confidence Interval | | | | | | |

# Supplementary Table 12: Regression output Iron vs CC-IMT in adults

| **Parameter** | **Coefficient** | **Standard Error** | **95% CI** | | **T.value** | **p-value** |
| --- | --- | --- | --- | --- | --- | --- |
|  |  |  | **Low** | **High** |  |  |
| **(Intercept)** | 0.648 | 0.061 | 0.528 | 0.769 | 10.571 | **<0.0001** |
| **Iron (μM)** |  |  |  |  |  |  |
| **<8.9** | 0.070 | 0.044 | -0.016 | 0.156 | 1.601 | 0.109 |
| **8.9-12.44** | -0.025 | 0.042 | -0.107 | 0.056 | -0.604 | 0.546 |
| **12.45-16.33** | 0.154 | 0.116 | -0.074 | 0.381 | 1.322 | 0.186 |
| **>16.33** | 0.107 | 0.173 | -0.233 | 0.446 | 0.618 | 0.537 |
| **Sex:F (ref=M)** | 0.012 | 0.061 | -0.108 | 0.133 | 0.202 | 0.840 |
| **Iron (μM): Females** |  |  |  |  |  |  |
| **<8.9** | -0.033 | 0.059 | -0.148 | 0.083 | -0.552 | 0.581 |
| **8.9-12.44** | -0.018 | 0.063 | -0.141 | 0.104 | -0.292 | 0.771 |
| **12.45-16.33** | -0.247 | 0.164 | -0.569 | 0.074 | -1.507 | 0.132 |
| **>16.33** | -0.244 | 0.226 | -0.686 | 0.198 | -1.081 | 0.280 |
| We fitted a linear mixed model (estimated using REML and nloptwrap optimizer) to predict CC-IMT with iron and sex (formula: imt ~ 1 + ns(iron, df = 4) * sex). The model included study as random effect (formula: ~1 \| study). Complete (unimputed) data was used. | | | | | | |

#

# Supplementary Table 13. Regression output Ferritin vs CC-IMT in children and adolescents

| **Parameter** | **Coefficient** | **95% CI** | **p-value** |
| --- | --- | --- | --- |
| **Ferritin (ng/mL)** |  |  |  |
| **<24** | 0.01 | -0.02, 0.05 | 0.46 |
| **24-37** | 0.01 | -0.04, 0.06 | >0.65 |
| **38-59** | 0.08 | -0.03, 0.18 | 0.16 |
| **>59** | 0.15 | -0.03, 0.33 | 0.099 |
| **Sex:F (ref=M)** | 0.01 | -0.03, 0.04 | 0.8 |
| **Ferritin (ng/mL): Females** |  |  |  |
| **<24** | -0.03 | -0.07, 0.01 | 0.11 |
| **24-37** | 0.02 | -0.05, 0.10 | 0.54 |
| **38-59** | -0.06 | -0.19, 0.06 | 0.31 |
| **>59** | -0.12 | -0.32, 0.08 | 0.26 |
| We fitted a linear mixed model (estimated using REML and nloptwrap optimizer) to predict CC-IMT with ferritin and sex (formula: imt ~ 1 + ns(ferritin, df = 4) * sex). The model included study as random effect (formula: ~1 \| study). CI = Confidence Interval | | | |

# Supplementary Table 14. Regression output TSAT vs CC-IMT in children and adolescents

| **Parameter** | **Coefficient** | **95% CI** | **p-value** |
| --- | --- | --- | --- |
| **TSAT (%)** |  |  |  |
| **<15** | 0.04 | -0.02, 0.11 | 0.2 |
| **15-19** | -0.04 | -0.12, 0.04 | 0.4 |
| **20-26** | 0.05 | -0.10, 0.21 | 0.5 |
| **>26** | 0.11 | -0.05, 0.26 | 0.2 |
| **Sex:F (ref=M)** | 0.03 | -0.06, 0.12 | 0.5 |
| **TSAT (%): Females** |  |  |  |
| **<15** | -0.04 | -0.13, 0.05 | 0.4 |
| **15-19** | 0.01 | -0.09, 0.12 | 0.8 |
| **20-26** | -0.11 | -0.32, 0.11 | 0.3 |
| **>26** | -0.09 | -0.27, 0.09 | 0.3 |
| We fitted a linear model (estimated using maximum likelihood) to predict CC-IMT with TSAT and sex (formula: imt ~ 1 + ns(TSAT, df = 4) * sex). Complete (unimputed) data was used. CI = Confidence Interval | | | |

# Supplementary Table 15. Regression output Iron vs CC-IMT in children and adolescents

| **Parameter** | **Coefficient** | **95% CI** | **p-value** |
| --- | --- | --- | --- |
| **Iron (μM)** |  |  |  |
| <8.9 | 0.001 | -0.05, 0.06 | 0.8 |
| 8.9-12.43 | -0.04 | -0.09, 0.01 | 0.13 |
| 12.44-16.34 | -0.08 | -0.2, 0.04 | 0.21 |
| >16.34 | 0.01 | -0.05, 0.07 | 0.73 |
| **Sex:F (ref=M)** | -0.01 | -0.09, 0.06 | 0.71 |
| **Iron (μM): Females** |  |  |  |
| <8.9 | 0.001 | -0.08, 0.08 | >0.9 |
| 8.9-12.43 | 0.02 | -0.06, 0.10 | 0.62 |
| 12.44-16.34 | 0.03 | -0.14, 0.21 | 0.71 |
| >16.34 | -0.02 | -0.12, 0.08 | 0.72 |
| We fitted a linear mixed model (estimated using REML and nloptwrap optimizer) to predict CC-IMT with iron and sex (formula: imt ~ 1 + ns(iron, df = 4) * sex). The model included study as random effect (formula: ~1 \| study). Complete (unimputed) data was used. | | | |

#

# Supplementary Table 16

| **Parameter** | **Coefficient** | **95% CI** | **p-value** |
| --- | --- | --- | --- |
| **Transferrin (g/L)** |  |  |  |
| **<251** | -0.02 | -0.15, 0.11 | 0.8 |
| **251-272** | -0.01 | -0.09, 0.08 | 0.9 |
| **273-297.75** | -0.04 | -0.31, 0.23 | 0.8 |
| **>297.75** | -0.02 | -0.13, 0.10 | 0.8 |
| **Sex:F (ref=M)** | 0.14 | -0.12, 0.41 | 0.3 |
| **Transferrin (g/L): Females** |  |  |  |
| **<251** | -0.15 | -0.41, 0.11 | 0.3 |
| **251-272** | -0.07 | -0.23, 0.09 | 0.4 |
| **273-297.75** | -0.34 | -0.88, 0.19 | 0.2 |
| **>297.75** | -0.17 | -0.35, 0.01 | 0.073 |
| We fitted a linear model (estimated using maximum likelihood) to predict CC-IMT with transferrin and sex (formula: imt ~ 1 + ns(transferrin, df = 4) * sex). Complete (unimputed) data was used. CI = Confidence Intervalice | | | |

| Supplementary Table 17. Multivariable regression analysis of ferritin and CC-IMT in nonsmokers (subgroup analysis) | | | | | | | |
| --- | --- | --- | --- | --- | --- | --- | --- |
| **Parameter** | **Estimate** | **95% CI** | | **Standard Error** | **Fraction of Missing Information (FMI)** | **Relative Increase in Variance (RIV)** | **p-value** |
|  |  | **Low** | **High** |  |  |  |  |
| **Intercept** | 0.614 | 0.407 | 0.821 | 0.106 | 0.540 | 0.956 | **<0.001** |
| **Ferritin (ng/mL)** | | | | | | | |
| **<67.06** | 0.023 | -0.165 | 0.211 | 0.096 | 0.543 | 0.966 | 0.814 |
| **67.06-131.9** | 0.121 | -0.036 | 0.279 | 0.080 | 0.625 | 1.321 | 0.156 |
| **132-232.07** | 0.140 | -0.294 | 0.575 | 0.222 | 0.579 | 1.103 | 0.536 |
| **>232.08** | 0.090 | -0.155 | 0.335 | 0.125 | 0.229 | 0.270 | 0.474 |
| **Sex:F (ref=M)** | -0.042 | -0.288 | 0.204 | 0.126 | 0.116 | 0.124 | 0.738 |
| **Ferritin (ng/mL): Females** | | | | | | | |
| **<67.06** | 0.009 | -0.236 | 0.254 | 0.125 | 0.129 | 0.140 | 0.945 |
| **67.06-131.9** | 0.043 | -0.178 | 0.264 | 0.113 | 0.238 | 0.281 | 0.704 |
| **132-232.07** | 0.244 | -0.310 | 0.797 | 0.282 | 0.113 | 0.121 | 0.389 |
| **>232.08** | 0.329 | -0.202 | 0.861 | 0.271 | 0.047 | 0.048 | 0.224 |
| **Other covariates** | | | | | | | |
| **Age** | 0.145 | 0.136 | 0.155 | 0.005 | 0.058 | 0.060 | **<0.001** |
| **Diabetes (ref= No Diabetes)** | 0.030 | 0.013 | 0.048 | 0.009 | 0.541 | 0.958 | **0.003** |
| **Creatinine** | -0.007 | -0.019 | 0.006 | 0.006 | 0.717 | 1.952 | 0.317 |
| **HDLc** | -0.006 | -0.011 | 0.000 | 0.003 | 0.140 | 0.152 | **0.036** |
| **LDLc** | 0.011 | 0.006 | 0.016 | 0.003 | 0.174 | 0.194 | **<0.001** |
| **Triacylglycerols** | -0.002 | -0.006 | 0.003 | 0.002 | 0.123 | 0.132 | 0.426 |
| **BMI** | 0.004 | -0.002 | 0.010 | 0.003 | 0.029 | 0.029 | 0.143 |
| **CRP** | 0.009 | 0.002 | 0.016 | 0.004 | 0.660 | 1.518 | **0.03** |
| **Hb** | 0.012 | 0.004 | 0.020 | 0.004 | 0.505 | 0.839 | **0.007** |
| **SBP** | 0.014 | 0.007 | 0.021 | 0.004 | 0.172 | 0.192 | **<0.001** |
| **DBP** | -0.008 | -0.015 | -0.002 | 0.004 | 0.148 | 0.163 | **0.018** |

| Supplementary Table 18. Multivariable regression analysis of transferrin and CC-IMT in nonsmokers (subgroup analysis) | | | | | | | |
| --- | --- | --- | --- | --- | --- | --- | --- |
| **Parameter** | **Estimate** | **95% CI** | | **Standard Error** | **Fraction of Missing Information (FMI)** | **Relative Increase in Variance (RIV)** | **p-value** |
|  |  | **Low** | **High** |  |  |  |  |
| **Intercept** | 0.692 | 0.561 | 0.823 | 0.067 | 0.283 | 0.350 | <0.001 |
| **Transferrin (g/L)** | | | | | | | |
| **< 196** | -0.055 | -0.178 | 0.068 | 0.063 | 0.456 | 0.701 | 0.391 |
| **197-231** | -0.021 | -0.107 | 0.065 | 0.044 | 0.207 | 0.238 | 0.634 |
| **232-259** | -0.121 | -0.423 | 0.182 | 0.154 | 0.460 | 0.709 | 0.443 |
| **>260** | -0.109 | -0.455 | 0.237 | 0.176 | 0.346 | 0.459 | 0.541 |
| **Sex:F (ref=M)** | 0.560 | 0.007 | 1.113 | 0.282 | 0.339 | 0.446 | 0.054 |
| **Transferrin (g/L): Females** | | | | | | | |
| **< 196** | -0.579 | -1.121 | -0.036 | 0.277 | 0.336 | 0.440 | 0.043 |
| **197-231** | -0.345 | -0.689 | -0.002 | 0.175 | 0.445 | 0.673 | 0.06 |
| **232-259** | -1.150 | -2.217 | -0.084 | 0.544 | 0.262 | 0.317 | 0.038 |
| **>260** | -0.234 | -0.641 | 0.172 | 0.207 | 0.086 | 0.090 | 0.259 |
| **Other covariates** | | | | | | | |
| **Age** | 0.146 | 0.136 | 0.155 | 0.005 | 0.031 | 0.032 | <0.001 |
| **Diabetes (ref= No Diabetes)** | 0.030 | 0.014 | 0.048 | 0.009 | 0.506 | 0.843 | 0.002 |
| **Creatinine** | -0.006 | -0.018 | 0.006 | 0.006 | 0.686 | 1.695 | 0.349 |
| **HDLc** | -0.005 | -0.011 | 0.000 | 0.003 | 0.122 | 0.132 | 0.046 |
| **LDLc** | 0.011 | 0.006 | 0.016 | 0.003 | 0.150 | 0.165 | <0.001 |
| **Triacylglycerols** | -0.001 | -0.005 | 0.004 | 0.002 | 0.119 | 0.128 | 0.709 |
| **BMI** | 0.005 | -0.001 | 0.011 | 0.003 | 0.070 | 0.072 | 0.12 |
| **CRP** | 0.010 | 0.004 | 0.017 | 0.003 | 0.614 | 1.264 | 0.007 |
| **Hb** | 0.012 | 0.004 | 0.021 | 0.004 | 0.590 | 1.151 | 0.012 |
| **SBP** | 0.014 | 0.007 | 0.020 | 0.004 | 0.169 | 0.188 | <0.001 |
| **DBP** | -0.008 | -0.015 | -0.002 | 0.004 | 0.139 | 0.151 | 0.018 |

| Supplementary Table 19. Multivariable regression analysis of ferritin and CC-IMT in normotensive individuals (subgroup analysis) | | | | | | | |
| --- | --- | --- | --- | --- | --- | --- | --- |
| **Parameter** | **Estimate** | **95% CI** | | **Standard Error** | **Fraction of Missing Information (FMI)** | **Relative Increase in Variance (RIV)** | **p-value** |
|  |  | **Low** | **High** |  |  |  |  |
| **Intercept** | 0.567 | 0.309 | 0.824 | 0.131 | 0.468 | 0.731 | **<0.001** |
| **Ferritin (ng/mL)** | | | | | | | |
| **<67.06** | 0.058 | -0.180 | 0.295 | 0.121 | 0.496 | 0.812 | 0.64 |
| **67.06-131.9** | 0.128 | -0.058 | 0.314 | 0.095 | 0.260 | 0.315 | 0.181 |
| **132-232.07** | 0.197 | -0.352 | 0.745 | 0.280 | 0.509 | 0.851 | 0.49 |
| **>232.08** | 0.006 | -0.350 | 0.363 | 0.182 | 0.099 | 0.105 | 0.971 |
| **Sex:F (ref=M)** | 0.214 | -0.443 | 0.871 | 0.335 | 0.797 | 2.962 | 0.543 |
| **Ferritin (ng/mL): Females** | | | | | | | |
| **<67.06** | -0.238 | -0.884 | 0.409 | 0.330 | 0.797 | 2.953 | 0.494 |
| **67.06-131.9** | -0.230 | -0.775 | 0.315 | 0.278 | 0.737 | 2.144 | 0.431 |
| **132-232.07** | 0.125 | -1.178 | 1.428 | 0.665 | 0.723 | 2.007 | 0.855 |
| **>232.08** | 1.220 | 0.373 | 2.067 | 0.432 | 0.075 | 0.079 | **0.005** |
| **Other covariates** | | | | | | | |
| **Age** | 0.137 | 0.122 | 0.152 | 0.008 | 0.009 | 0.009 | **<0.001** |
| **Diabetes (ref= No Diabetes)** | 0.011 | -0.010 | 0.032 | 0.011 | 0.241 | 0.286 | 0.315 |
| **Creatinine** | -0.001 | -0.017 | 0.015 | 0.008 | 0.578 | 1.100 | 0.894 |
| **HDLc** | -0.007 | -0.016 | 0.003 | 0.005 | 0.475 | 0.751 | 0.17 |
| **LDLc** | 0.006 | -0.001 | 0.013 | 0.004 | 0.269 | 0.328 | 0.109 |
| **Triacylglycerols** | 0.003 | -0.003 | 0.009 | 0.003 | 0.114 | 0.122 | 0.358 |
| **BMI** | 0.006 | -0.001 | 0.014 | 0.004 | 0.006 | 0.006 | 0.106 |
| **CRP** | 0.013 | 0.004 | 0.022 | 0.005 | 0.600 | 1.198 | **0.013** |
| **Hb** | 0.005 | -0.005 | 0.016 | 0.005 | 0.487 | 0.786 | 0.338 |
| **SBP** | 0.032 | 0.021 | 0.042 | 0.005 | 0.139 | 0.151 | **<0.001** |
| **DBP** | -0.016 | -0.025 | -0.006 | 0.005 | 0.204 | 0.234 | **0.002** |

| Supplementary Table 20. Multivariable regression analysis of transferrin and CC-IMT in normotensive individuals (subgroup analysis) | | | | | | | |
| --- | --- | --- | --- | --- | --- | --- | --- |
| **Parameter** | **Estimate** | **95% CI** | | **Standard Error** | **Fraction of Missing Information (FMI)** | **Relative Increase in Variance (RIV)** | **p-value** |
|  |  | **Low** | **High** |  |  |  |  |
| **Intercept** | 0.628 | 0.399 | 0.857 | 0.117 | 0.443 | 0.668 | **<0.001** |
| **Transferrin (g/L)** | | | | | | | |
| **< 196** | -0.007 | -0.244 | 0.230 | 0.121 | 0.598 | 1.185 | 0.953 |
| **197-231** | 0.018 | -0.109 | 0.145 | 0.065 | 0.147 | 0.161 | 0.783 |
| **232-259** | -0.163 | -0.590 | 0.264 | 0.218 | 0.188 | 0.212 | 0.456 |
| **>260** | -0.392 | -0.941 | 0.157 | 0.280 | 0.227 | 0.265 | 0.165 |
| **Sex:F (ref=M)** | 0.595 | 0.183 | 1.007 | 0.210 | 0.438 | 0.654 | **0.009** |
| **Transferrin (g/L): Females** | | | | | | | |
| **< 196** | -0.615 | -1.027 | -0.204 | 0.210 | 0.464 | 0.720 | **0.007** |
| **197-231** | -0.341 | -0.624 | -0.058 | 0.144 | 0.505 | 0.838 | **0.029** |
| **232-259** | -1.232 | -2.096 | -0.369 | 0.441 | 0.396 | 0.558 | **0.009** |
| **>260** | -0.241 | -1.051 | 0.569 | 0.413 | 0.351 | 0.469 | 0.563 |
| **Other covariates** | | | | | | | |
| **Age** | 0.137 | 0.122 | 0.152 | 0.008 | 0.053 | 0.055 | **<0.001** |
| **Diabetes (ref= No Diabetes)** | 0.012 | -0.008 | 0.033 | 0.011 | 0.213 | 0.246 | 0.247 |
| **Creatinine** | -0.002 | -0.017 | 0.012 | 0.007 | 0.500 | 0.825 | 0.741 |
| **HDLc** | -0.006 | -0.016 | 0.003 | 0.005 | 0.448 | 0.679 | 0.18 |
| **LDLc** | 0.007 | 0.000 | 0.014 | 0.004 | 0.231 | 0.271 | 0.066 |
| **Triacylglycerols** | 0.003 | -0.003 | 0.010 | 0.003 | 0.111 | 0.118 | 0.29 |
| **BMI** | 0.006 | -0.002 | 0.014 | 0.004 | 0.073 | 0.076 | 0.115 |
| **CRP** | 0.013 | 0.004 | 0.023 | 0.005 | 0.678 | 1.639 | **0.02** |
| **Hb** | 0.006 | -0.005 | 0.017 | 0.006 | 0.492 | 0.799 | 0.3 |
| **SBP** | 0.032 | 0.022 | 0.042 | 0.005 | 0.147 | 0.162 | **<0.001** |
| **DBP** | -0.016 | -0.025 | -0.006 | 0.005 | 0.154 | 0.170 | **0.001** |

References

1. R Core Team. R: A Language and Environment for Statistical Computing. R Foundation for Statistical Computing; 2023. <https://www.R-project.org/>

2. Bååth R. beepr: Easily Play Notification Sounds on Any Platform.; 2018. <https://CRAN.R-project.org/package=beepr>

3. Patil I. broomExtra: Enhancements for “broom” and “easystats” Package Families.; 2023.

4. Bivand R. classInt: Choose Univariate Class Intervals.; 2023. <https://CRAN.R-project.org/package=classInt>

5. Rudis B, Muir C. docxtractr: Extract Data Tables and Comments from “Microsoft” “Word” Documents.; 2020. <https://CRAN.R-project.org/package=docxtractr>

6. Xie Y, Cheng J, Tan X. DT: A Wrapper of the JavaScript Library “DataTables”.; 2023. <https://CRAN.R-project.org/package=DT>

7. Lüdecke D, Ben-Shachar MS, Patil I, Wiernik BM, Makowski D. easystats: Framework for easy statistical modeling, visualization, and reporting. CRAN. Published online 2022. <https://easystats.github.io/easystats/>

8. Aden-Buie G, Sievert C, Iannone R, Allaire J, Borges B. flexdashboard: R Markdown Format for Flexible Dashboards.; 2023. <https://CRAN.R-project.org/package=flexdashboard>

9. Gohel D, Skintzos P. flextable: Functions for Tabular Reporting.; 2023. <https://CRAN.R-project.org/package=flextable>

10. R Core Team. foreign: Read Data Stored by “Minitab,” “S,” “SAS,” “SPSS,” “Stata,” “Systat,” “Weka,” “dBase,” ...; 2023. <https://CRAN.R-project.org/package=foreign>

11. Ren K, Russell K. formattable: Create “Formattable” Data Structures.; 2021. <https://CRAN.R-project.org/package=formattable>

12. Kay M. ggdist: Visualizations of Distributions and Uncertainty.; 2023. doi:[10.5281/zenodo.3879620](https://doi.org/10.5281/zenodo.3879620)

13. Tiedemann F. gghalves: Compose Half-Half Plots Using Your Favourite Geoms.; 2022. <https://CRAN.R-project.org/package=gghalves>

14. Oberman H. ggmice: Visualizations for “mice” with “ggplot2”.; 2023. <https://CRAN.R-project.org/package=ggmice>

15. Kelkhoff D. ggpackets: Package Plot Layers for Easier Portability and Modularization.; 2022. <https://CRAN.R-project.org/package=ggpackets>

16. Yu G. ggplotify: Convert Plot to “grob” or “ggplot” Object.; 2023. <https://CRAN.R-project.org/package=ggplotify>

17. Kassambara A. ggpubr: “ggplot2” Based Publication Ready Plots.; 2023. <https://CRAN.R-project.org/package=ggpubr>

18. Wilke CO, Wiernik BM. ggtext: Improved Text Rendering Support for “ggplot2”.; 2022. <https://CRAN.R-project.org/package=ggtext>

19. Auguie B. gridExtra: Miscellaneous Functions for “Grid” Graphics.; 2017. <https://CRAN.R-project.org/package=gridExtra>

20. Iannone R, Cheng J, Schloerke B, Hughes E, Lauer A, Seo J. gt: Easily Create Presentation-Ready Display Tables.; 2023. <https://CRAN.R-project.org/package=gt>

21. Sjoberg DD, Whiting K, Curry M, Lavery JA, Larmarange J. Reproducible summary tables with the gtsummary package. The R Journal. 2021;13:570-580. doi:[10.32614/RJ-2021-053](https://doi.org/10.32614/RJ-2021-053)

22. Müller K. here: A Simpler Way to Find Your Files.; 2020. <https://CRAN.R-project.org/package=here>

23. Firke S. janitor: Simple Tools for Examining and Cleaning Dirty Data.; 2023. <https://CRAN.R-project.org/package=janitor>

24. Quartagno M, Carpenter J. jomo: A Package for Multilevel Joint Modelling Multiple Imputation.; 2023. <https://CRAN.R-project.org/package=jomo>

25. Zhu H. kableExtra: Construct Complex Table with “kable” and Pipe Syntax.; 2021. <https://CRAN.R-project.org/package=kableExtra>

26. Xie Y. knitr: A General-Purpose Package for Dynamic Report Generation in r.; 2023. <https://yihui.org/knitr/>

27. Larmarange J. labelled: Manipulating Labelled Data.; 2023. <https://CRAN.R-project.org/package=labelled>

28. Sarkar D. Lattice: Multivariate Data Visualization with r. Springer; 2008. <http://lmdvr.r-forge.r-project.org>

29. Bates D, Mächler M, Bolker B, Walker S. Fitting linear mixed-effects models using lme4. Journal of Statistical Software. 2015;67(1):1-48. doi:[10.18637/jss.v067.i01](https://doi.org/10.18637/jss.v067.i01)

30. Kuznetsova A, Brockhoff PB, Christensen RHB. lmerTest package: Tests in linear mixed effects models. Journal of Statistical Software. 2017;82(13):1-26. doi:[10.18637/jss.v082.i13](https://doi.org/10.18637/jss.v082.i13)

31. van Buuren S, Groothuis-Oudshoorn K. mice: Multivariate imputation by chained equations in r. Journal of Statistical Software. 2011;45(3):1-67. doi:[10.18637/jss.v045.i03](https://doi.org/10.18637/jss.v045.i03)

32. Grund S, Robitzsch A, Luedtke O. Mitml: Tools for Multiple Imputation in Multilevel Modeling.; 2023. <https://CRAN.R-project.org/package=mitml>

33. Lumley T. Mitools: Tools for Multiple Imputation of Missing Data.; 2019. <https://CRAN.R-project.org/package=mitools>

34. Tierney N, Cook D. Expanding tidy data principles to facilitate missing data exploration, visualization and assessment of imputations. Journal of Statistical Software. 2023;105(7):1-31. doi:[10.18637/jss.v105.i07](https://doi.org/10.18637/jss.v105.i07)

35. Gohel D. officer: Manipulation of Microsoft Word and PowerPoint Documents.; 2023. <https://CRAN.R-project.org/package=officer>

36. Rinker TW, Kurkiewicz D. pacman: Package Management for R.; 2018. <http://github.com/trinker/pacman>

37. Zhao JH, Schafer JL. pan: Multiple Imputation for Multivariate Panel or Clustered Data.; 2023.

38. Neuwirth E. RColorBrewer: ColorBrewer Palettes.; 2022. <https://CRAN.R-project.org/package=RColorBrewer>

39. Ushey K, Wickham H. renv: Project Environments.; 2023. <https://CRAN.R-project.org/package=renv>

40. Allaire J, Xie Y, Dervieux C, et al. rmarkdown: Dynamic Documents for r.; 2023. <https://github.com/rstudio/rmarkdown>

41. Atkins A, Allen T, Wickham H, McPherson J, Allaire J. rsconnect: Deploy Docs, Apps, and APIs to “Posit Connect,” “shinyapps.io,” and “RPubs”.; 2023. <https://CRAN.R-project.org/package=rsconnect>

42. Gohel D. rvg: R Graphics Devices for “Office” Vector Graphics Output.; 2023. <https://CRAN.R-project.org/package=rvg>

43. South A. rworldmap: A new R package for mapping global data. The R Journal. 2011;3(1):35-43. doi:[10.32614/RJ-2011-006](https://doi.org/10.32614/RJ-2011-006)

44. Chang W, Cheng J, Allaire J, et al. shiny: Web Application Framework for r.; 2023. <https://CRAN.R-project.org/package=shiny>

45. Wickham H, Averick M, Bryan J, et al. Welcome to the tidyverse. Journal of Open Source Software. 2019;4(43):1686. doi:[10.21105/joss.01686](https://doi.org/10.21105/joss.01686)

46. Ooms J. writexl: Export Data Frames to Excel “xlsx” Format.; 2023. <https://CRAN.R-project.org/package=writexl>

47. Pawlak K, Pawlak D, Mysliwiec M. Long-term erythropoietin therapy decreases CC-chemokine levels and intima-media thickness in hemodialyzed patients. Am J Nephrol. 2006;26(5):497-502. doi:[10.1159/000097269](https://doi.org/10.1159/000097269)

48. Anderson TJ, Charbonneau F, Title LM, et al. Microvascular function predicts cardiovascular events in primary prevention: Long-term results from the firefighters and their endothelium (FATE) study. Circulation. 2011;123(2):163-169. doi:[10.1161/CIRCULATIONAHA.110.953653](https://doi.org/10.1161/CIRCULATIONAHA.110.953653)

49. Valenti L, Swinkels DW, Burdick L, et al. Serum ferritin levels are associated with vascular damage in patients with nonalcoholic fatty liver disease. Nutr Metab Cardiovasc Dis. 2011;21(8):568-575. doi:[10.1016/j.numecd.2010.01.003](https://doi.org/10.1016/j.numecd.2010.01.003)

50. Dvorakova HM, Szitanyi P, Dvorak P, et al. Determinants of premature atherosclerosis in children with end-stage renal disease. Physiol Res. 2012;61(1):53-61. doi:[10.33549/physiolres.932127](https://doi.org/10.33549/physiolres.932127)

51. Fernandez-Real JM, Puig J, Serrano M, et al. Iron and obesity status-associated insulin resistance influence circulating fibroblast-growth factor-23 concentrations. PLoS One. 2013;8(3):e58961. doi:[10.1371/journal.pone.0058961](https://doi.org/10.1371/journal.pone.0058961)

52. Formanowicz D, Wanic-Kossowska M, Pawliczak E, Radom M, Formanowicz P. Usefulness of serum interleukin-18 in predicting cardiovascular mortality in patients with chronic kidney diseasesystems and clinical approach. Sci Rep. 2015;5:18332. doi:[10.1038/srep18332](https://doi.org/10.1038/srep18332)

53. Arroyo D, Betriu A, Martinez-Alonso M, Vidal T, Valdivielso JM, Fernández E. Observational multicenter study to evaluate the prevalence and prognosis of subclinical atheromatosis in a Spanish chronic kidney disease cohort: baseline data from the NEFRONA study. BMC Nephrology. 2014;15(1):168. doi:[10.1186/1471-2369-15-168](https://doi.org/10.1186/1471-2369-15-168)

54. Prats-Puig A, Moreno M, Carreras-Badosa G, et al. Serum ferritin relates to carotid intima-media thickness in offspring of fathers with higher serum ferritin levels. Arterioscler Thromb Vasc Biol. 2016;36(1):174-180. doi:[10.1161/ATVBAHA.115.306396](https://doi.org/10.1161/ATVBAHA.115.306396)

55. Galesloot TE, Holewijn S, Kiemeney LA, Graaf J de, Vermeulen SH, Swinkels DW. Serum hepcidin is associated with presence of plaque in postmenopausal women of a general population. Arterioscler Thromb Vasc Biol. 2014;34(2):446-456. doi:[10.1161/ATVBAHA.113.302381](https://doi.org/10.1161/ATVBAHA.113.302381)

56. Risko P, Platenik J, Buchal R, Potockova J, Kraml PJ. The labile iron pool in monocytes reflects the activity of the atherosclerotic process in men with chronic cardiovascular disease. Physiol Res. 2017;66(1):49-61. doi:[10.33549/physiolres.933368](https://doi.org/10.33549/physiolres.933368)

57. Risko P, Platenik J, Buchal R, Potockova J, Kraml PJ. Long-term donors versus non-donor men: Iron metabolism and the atherosclerotic process. Atherosclerosis. 2018;272:14-20. doi:[10.1016/j.atherosclerosis.2018.03.009](https://doi.org/10.1016/j.atherosclerosis.2018.03.009)

58. Syrovatka P, Kraml P, Hulikova K, et al. Iron stores are associated with asymptomatic atherosclerosis in healthy men of primary prevention. Eur J Clin Invest. 2011;41(8):846-853. doi:[10.1111/j.1365-2362.2011.02474.x](https://doi.org/10.1111/j.1365-2362.2011.02474.x)

59. Agarvas AR, Kopf S, Thalmann P, Fernández-Real JM, Nawroth P, Muckenthaler MU. Markers of systemic iron status show sex-specific differences in peripheral artery disease: A cross-sectional analysis HEIST-DiC and NHANES participants. medRxiv. Published online 2023:2023.08.21.23293418. doi:[10.1101/2023.08.21.23293418](https://doi.org/10.1101/2023.08.21.23293418)

60. Bernar B, Gande N, Stock KA, et al. The tyrolean early vascular ageing-study (EVA-tyrol): Study protocol for a non-randomized controlled trial : Effect of a cardiovascular health promotion program in youth, a prospective cohort study. BMC Cardiovasc Disord. 2020;20(1):59. doi:[10.1186/s12872-020-01357-9](https://doi.org/10.1186/s12872-020-01357-9)

61. Kusic Milicevic J, Vidakovic R, Markovic R, et al. Cardiovascular risk assessment and coronary artery calcification burden in asymptomatic patients in the initial years of hemodialysis. Ther Apher Dial. 2022;26(1):64-70. doi:[10.1111/1744-9987.13641](https://doi.org/10.1111/1744-9987.13641)

62. Asicioglu E, Kahveci A, Arikan H, Koc M, Tuglular S, Ozener C. Fibroblast growth factor-23 levels are associated with uric acid but not carotid intima media thickness in renal transplant recipients. Transplant Proc. 2014;46(1):180-183. doi:[10.1016/j.transproceed.2013.10.043](https://doi.org/10.1016/j.transproceed.2013.10.043)

63. Hahalis G, Kremastinos DT, Terzis G, et al. Global vasomotor dysfunction and accelerated vascular aging in beta-thalassemia major. Atherosclerosis. 2008;198(2):448-457. doi:[10.1016/j.atherosclerosis.2007.09.030](https://doi.org/10.1016/j.atherosclerosis.2007.09.030)

64. Altamura C, Squitti R, Pasqualetti P, et al. Ceruloplasmin/transferrin system is related to clinical status in acute stroke. Stroke. 2009;40(4):1282-1288. doi:[10.1161/STROKEAHA.108.536714](https://doi.org/10.1161/STROKEAHA.108.536714)

65. Lai S, Dimko M, Galani A, et al. Early markers of cardiovascular risk in chronic kidney disease. Ren Fail. 2015;37(2):254-261. doi:[10.3109/0886022X.2014.982489](https://doi.org/10.3109/0886022X.2014.982489)

66. Merchant RH, Chate S, Ahmed J, Ahmad N, Karnik A, Jankaria B. Evaluation of carotid artery dynamics & correlation with cardiac & hepatic iron in beta-thalassaemia patients. Indian J Med Res. 2016;143(4):443-448. doi:[10.4103/0971-5916.184302](https://doi.org/10.4103/0971-5916.184302)

67. Abaza SE, Abdel-Salam A, Baz AA, Mohamed AA. Carotid doppler ultrasonography as a screening tool of early atherosclerotic changes in children and young adults with beta-thalassemia major. J Ultrasound. 2017;20(4):301-308. doi:[10.1007/s40477-017-0264-3](https://doi.org/10.1007/s40477-017-0264-3)
