# Supplementary material for: Ferritin and transferrin predict common carotid intima-media thickness in females: a machine-learning informed individual participant data meta-analysis
Source: BMC Cardiovasc Disord. 2026 Apr 14;26:360. doi: 10.1186/s12872-026-05796-8 (PMC13123155; doi:10.1186/s12872-026-05796-8)

Supplementary Material 2. Contains Supplementary Figures 1-7

Supplementary Figure 1. Scheme used for data requests in the study

Supplementary Figure 2. Rain cloud plots of serum iron categorized by age group and sex

Supplementary Figure 3. Rain cloud plots of serum ferritin categorized by age group and sex

Supplementary Figure 4. Rain cloud plots of serum transferrin categorized by age group and sex

Supplementary Figure 5. Rain cloud plots of serum TSAT categorized by age group and sex

Supplementary Figure 6. Rain cloud plots of CC-IMT categorized by age group and sex

Supplementary Figure 7. Figure showing the pattern of co-occurrences and instances of missing values in the dataset

Supplementary Figure 1

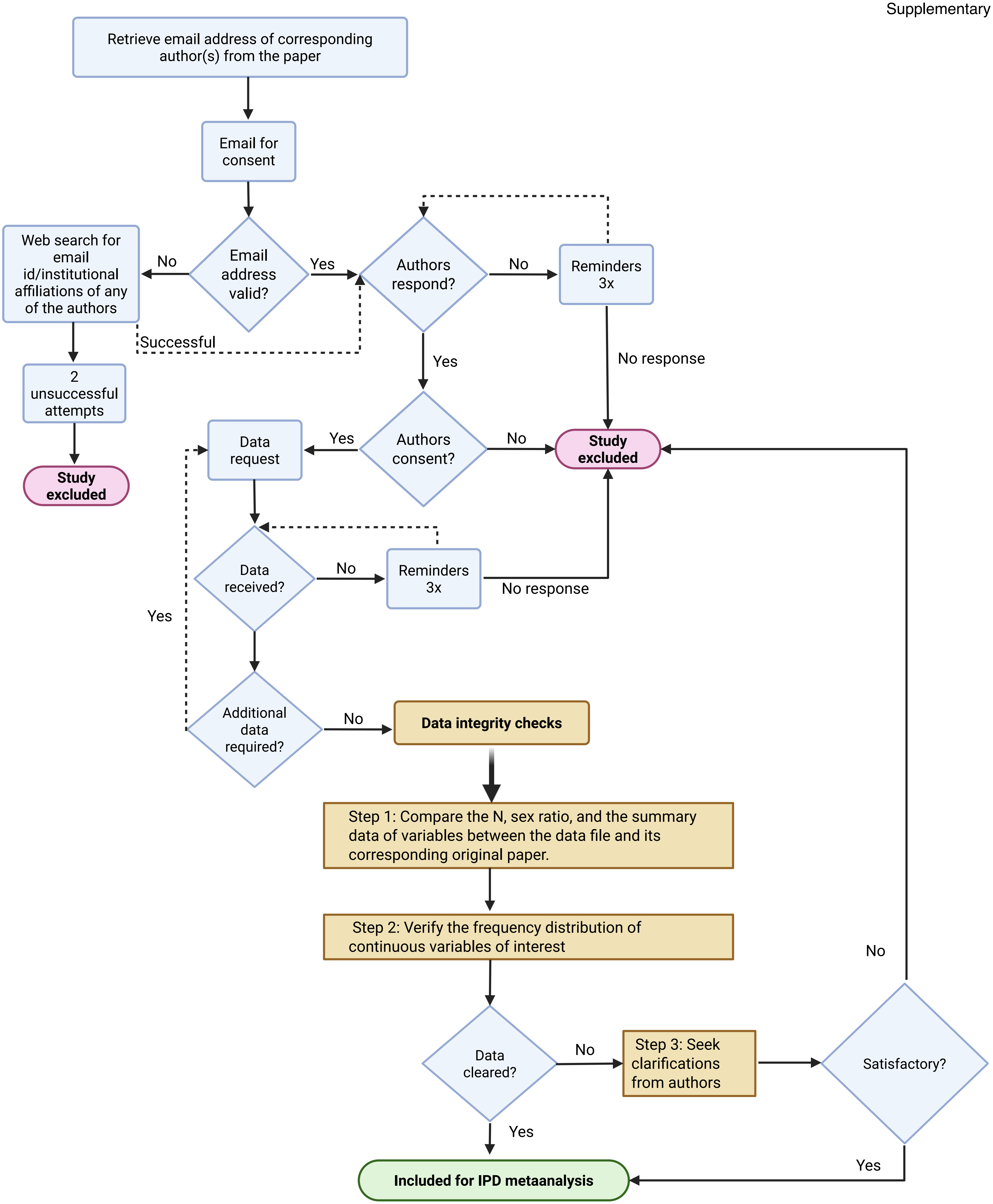

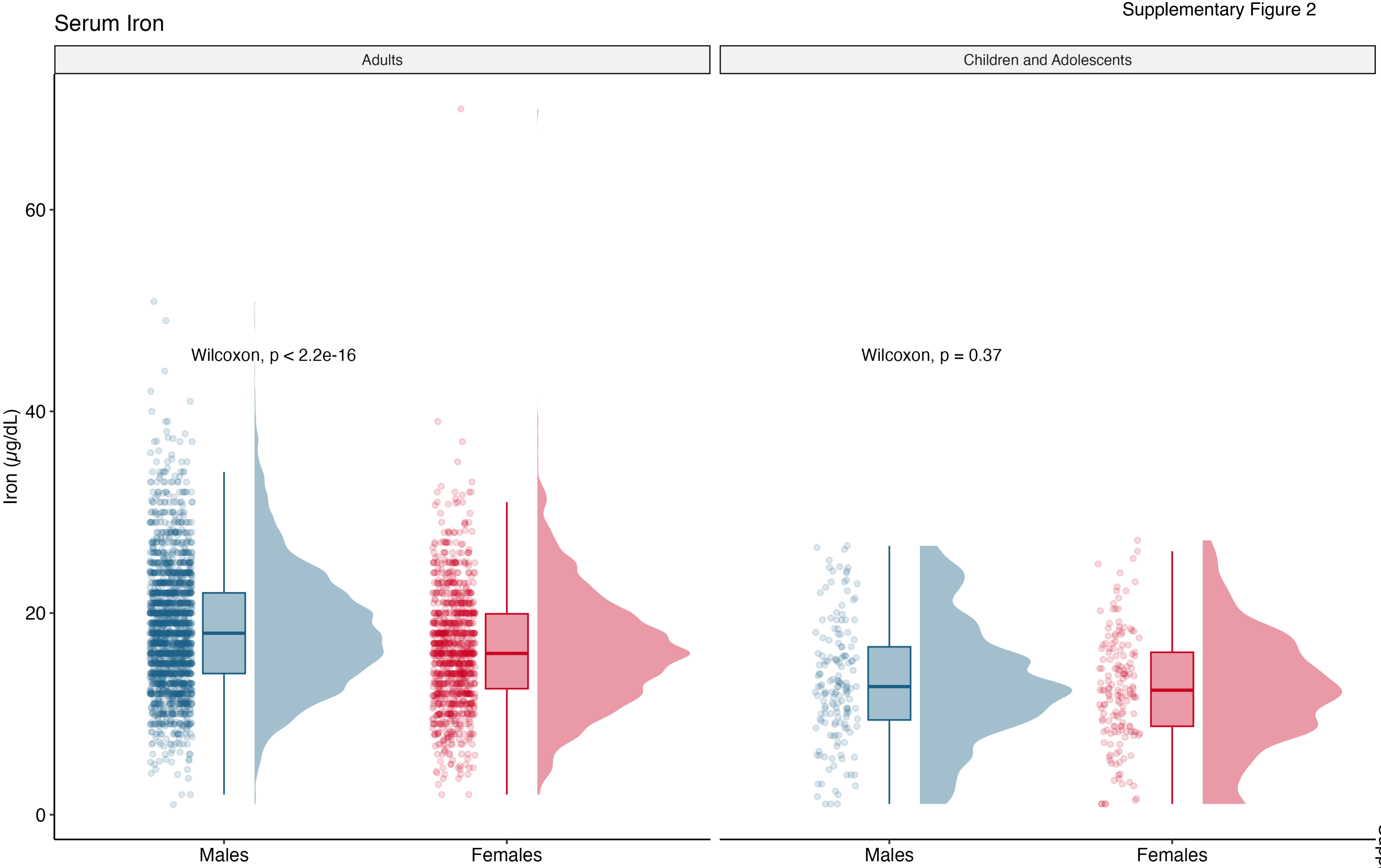
Serum Iron
Adults
Children and Adolescents
Iron (μg/dL)
60
40
20
0
Wilcoxon, p < 2.2e-16
Wilcoxon, p = 0.37
Males
Females
Males
Females

Supplementary Figure 3

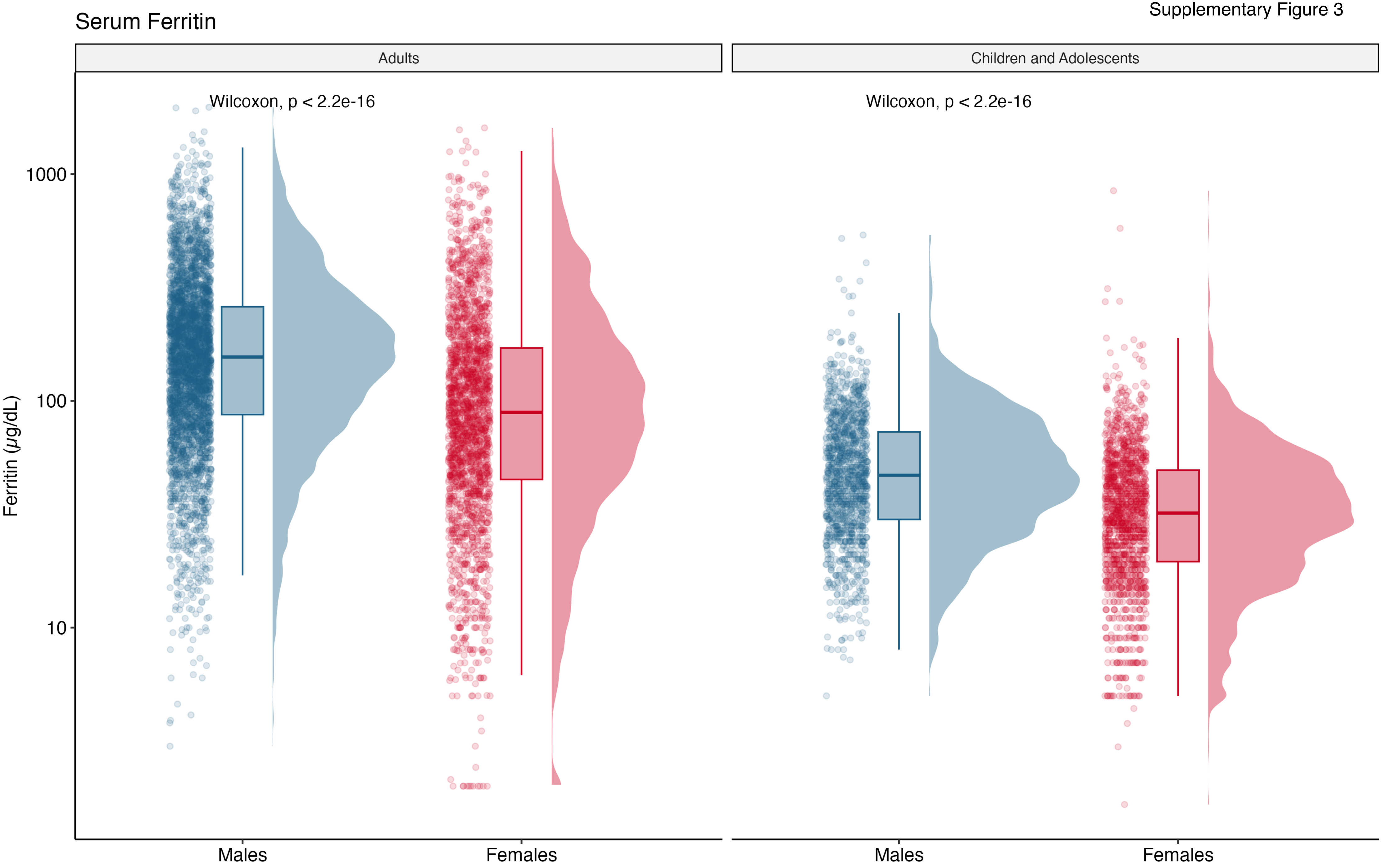

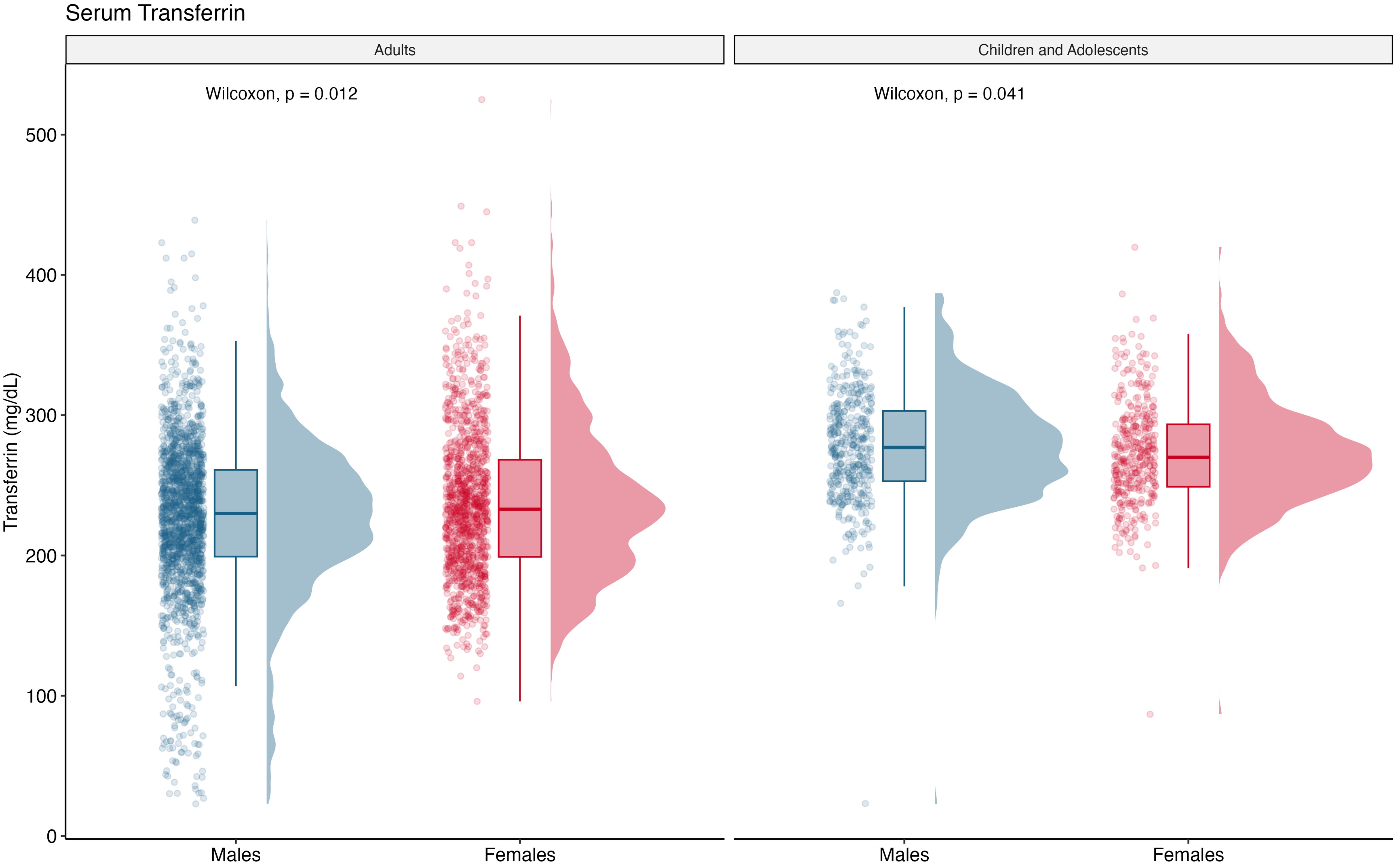
Serum Transferrin
Adults
Children and Adolescents
Wilcoxon, p = 0.012
Wilcoxon, p = 0.041
Transferrin (mg/dL)
0
100
200
300
400
500
Males
Females
Males
Females

Supplementary Figure 5

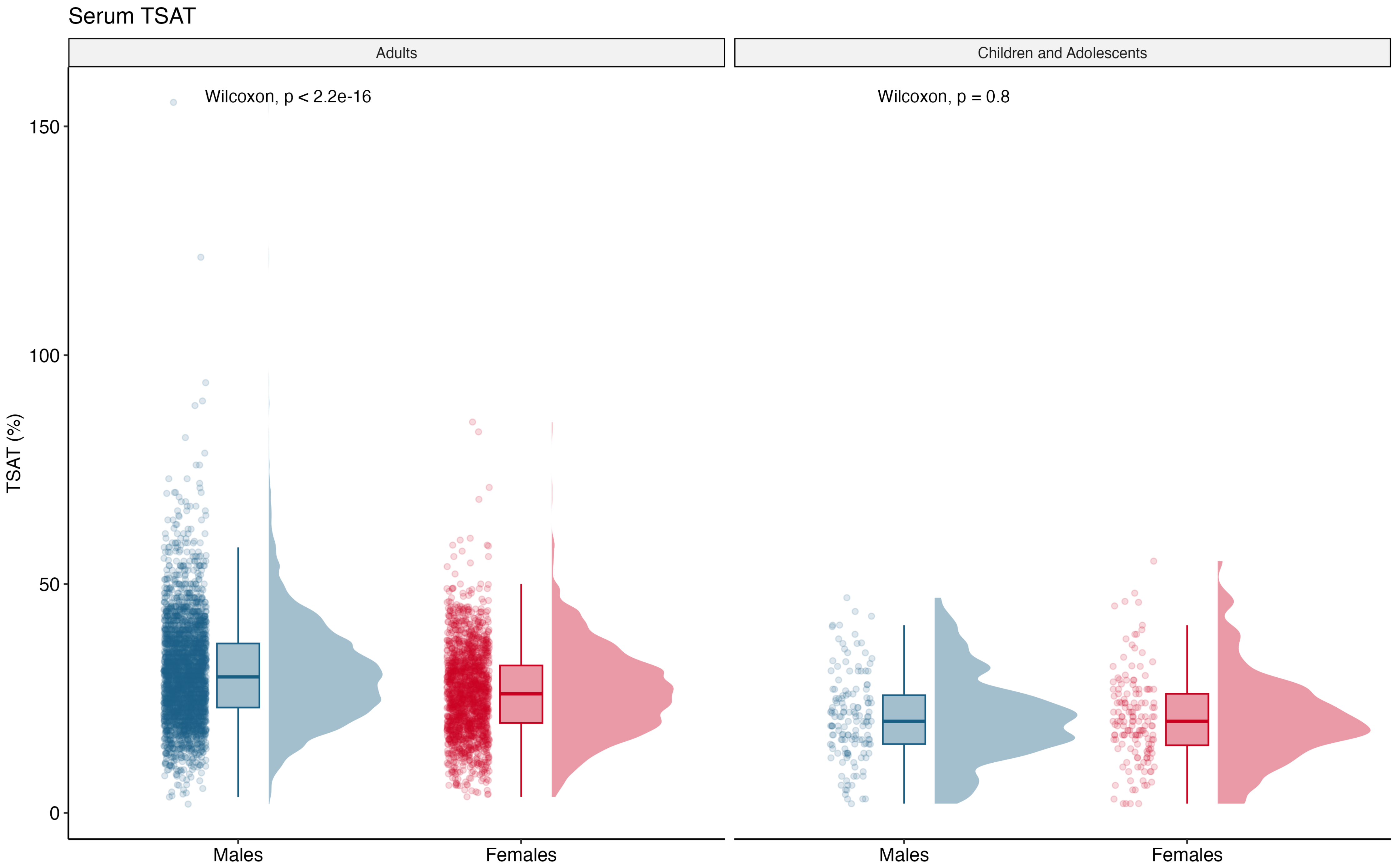

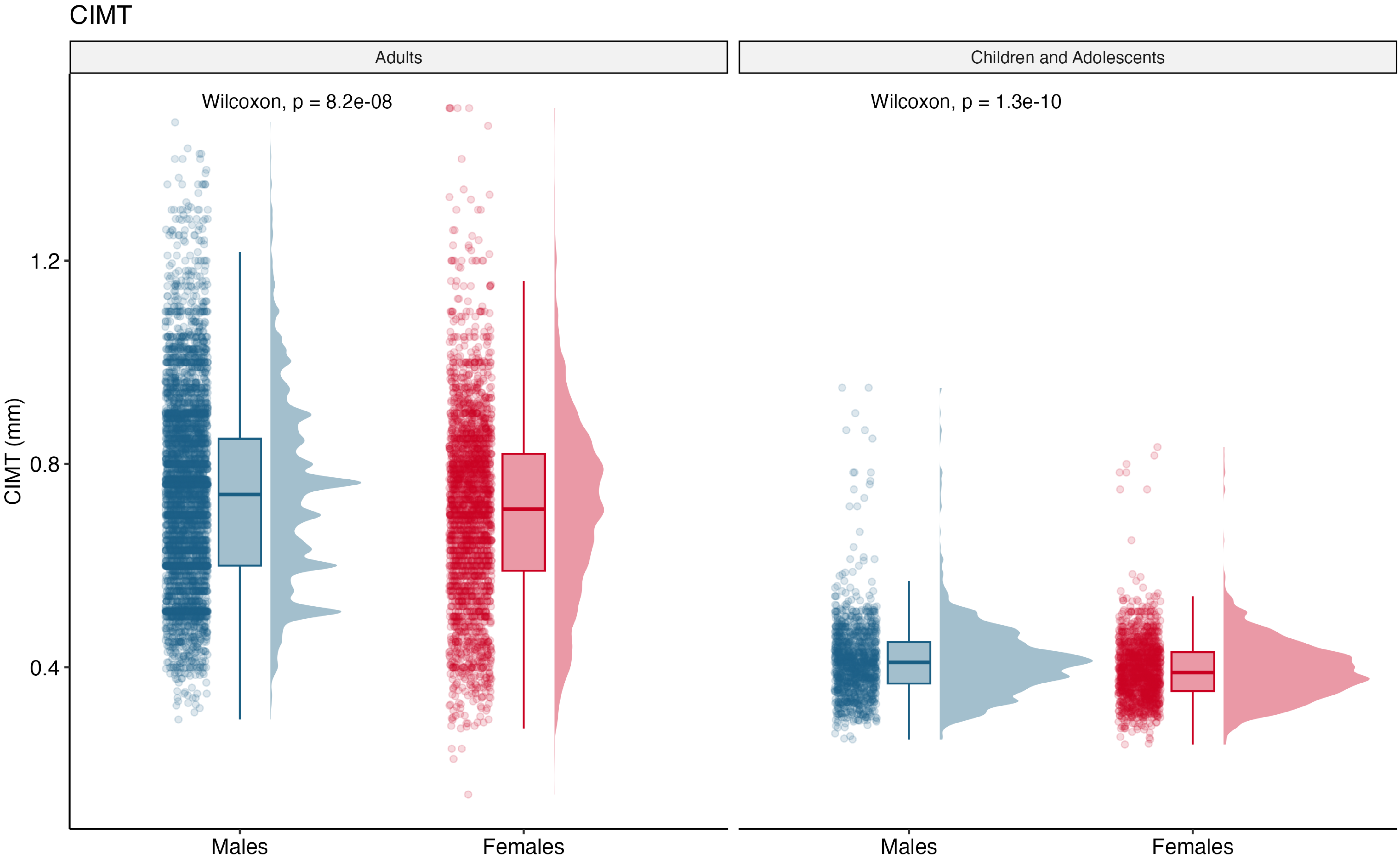
CIMT
Adults
Children and Adolescents
Wilcoxon, p = 8.2e-08
Wilcoxon, p = 1.3e-10
CIMT (mm)
1.2
0.8
0.4
Males
Females
Males
Females

Supplementary Figure 7

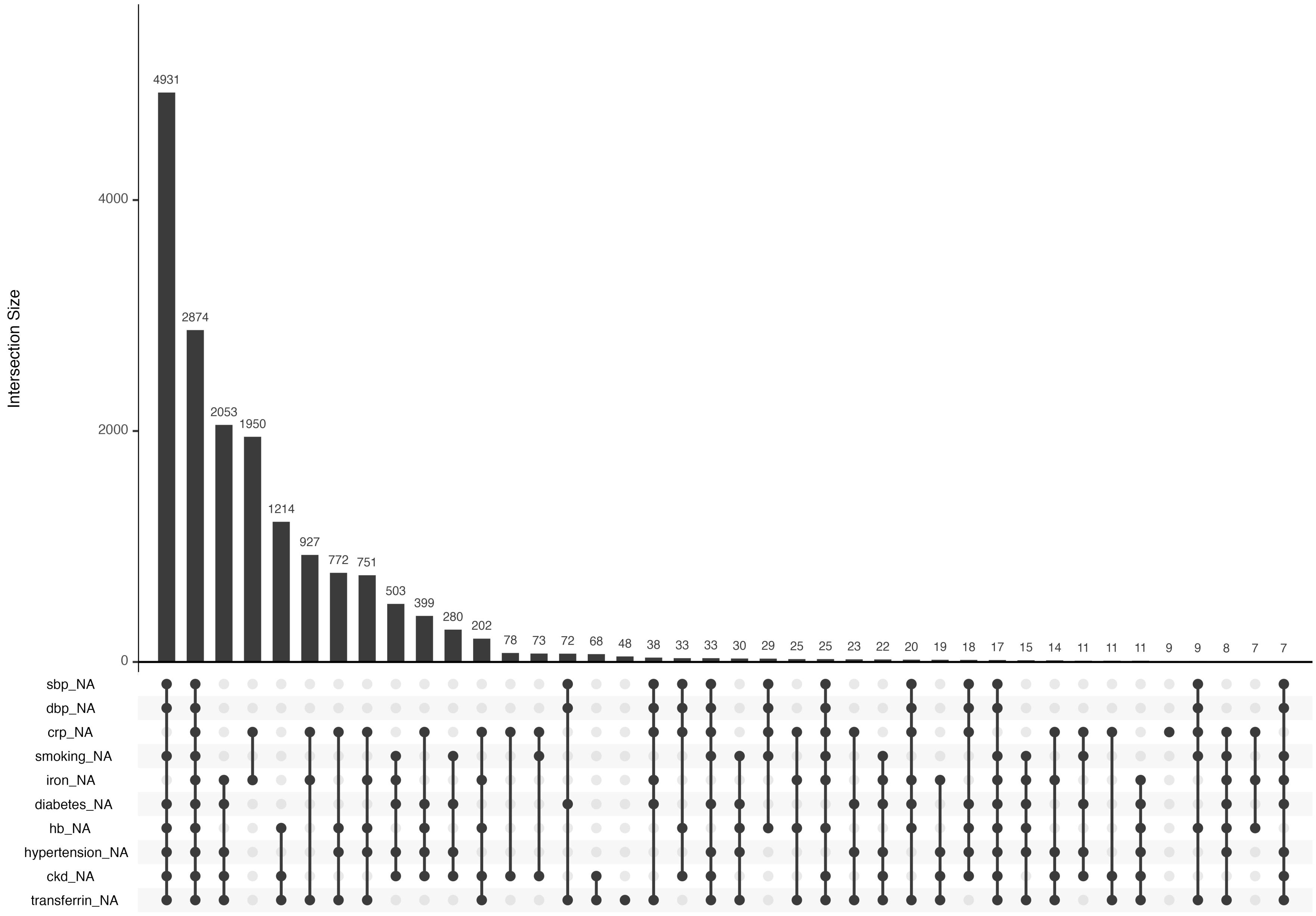

Supplement: Supplementary file 2 — Supplementary Material 2: Contains Supplementary Figures 1-7 [file 12872_2026_5796_MOESM2_ESM.pdf]
