# Supplementary material for: Ferritin and transferrin predict common carotid intima-media thickness in females: a machine-learning informed individual participant data meta-analysis
Source: BMC Cardiovasc Disord. 2026 Apr 14;26:360. doi: 10.1186/s12872-026-05796-8 (PMC13123155; doi:10.1186/s12872-026-05796-8)
Supplement: Supplementary file 9 — Supplementary Material 9: Pseudocode of the Machine learning pipeline used for IMT prediction [file 12872_2026_5796_MOESM9_ESM.pdf]

**Supplemetary file 3.**
**Machine Learning Pipeline for IMT Prediction.**

Input:
df ← dataset with predictors and target variable (imt)
study ← grouping variable for stratified splitting

Procedure:

1. Data Splitting:
   - Split df into training (75%) and test (25%) sets
   - Stratify by 'study' to preserve study-level representation

2. Preprocessing:
   - Define preprocessing recipe:
     a. Treat 'study' as identifier (exclude from predictors)
     b. Impute missing predictor values using bagged tree imputation
     c. Center and scale all numeric predictors (z-score normalization)
   - Apply preprocessing to training data

3. Model Specification:
   - Define XGBoost regression model with tunable hyperparameters:
     • Number of trees (trees)
     • Tree depth (tree_depth)
     • Learning rate (learn_rate)

4. Hyperparameter Tuning:
   - Generate space-filling grid of 15 hyperparameter combinations
   - Perform 10-fold cross-validation on training set
   - For each fold:
     a. Train model on 9 folds
     b. Evaluate RMSE on the held-out fold
   - Select hyperparameters with lowest mean RMSE

5. Model Fitting and Evaluation:
   - Refit final model on full training data using best parameters
   - Evaluate performance on held-out test data:
     • Compute RMSE and $R^2$ metrics
     • Store predictions for downstream analysis

6. Model Interpretation:
   - Compute variable importance (VIP) for top 20 predictors
   - Compute SHAP values for all predictors:
     • Generate SHAP importance and dependence plots

Output:
   - Final trained model
   - Performance metrics (RMSE, $R^2$)
   - VIP and SHAP visualizations for feature interpretability
